# Supplementary material for: RosettaAntibodyDesign (RAbD): A general framework for computational antibody design
Source: PLoS Comput Biol. 2018 Apr 27;14(4):e1006112. doi: 10.1371/journal.pcbi.1006112 (PMC5942852; doi:10.1371/journal.pcbi.1006112)
Supplement: S1 Text — This file contains supplementary text describing the methods and consists of the following 4 sections: Rosetta CommandsDihedral, Epitope, and Paratope ConstraintsOutlier ControlAntibody Feature Analysis (PDF) [file pcbi.1006112.s001.pdf]

Supplemental Methods
1. Rosetta Commands
2. Dihedral, Epitope, and Paratope Constraints
3. Outlier Control
4. Antibody Feature Analysis

1. Rosetta Commands

For the benchmarking set, full commands and CDR Instruction File contents are given below. Note that some options are the default and do not need to be specified, but are given in the file explicitly anyway. To prepare the structures for design, they are relaxed with the Rosetta force field using the FastRelax protocol with these commands:

Command:

```
relax.mpi.linuxgccrelease –l PDBLIST.txt –nstruct 10
@pareto_optimal_flags.txt
```

Contents of ***pareto_optimal_flags.txt*** *file:*

```
-no_optH false
-flip_HNQ
-use_input_sc
-constrain_relax_to_start_coords
-relax:ramp_constraints false
-relax:coord_constrain_sidechains
-ignore_unrecognized_res
-ignore_zero_occupancy false
-pdb_comments
-ex1
-ex2
-out:pdb_gz
-other_pose_to_scorefile
-scorefile_format json
-jd2:delete_old_poses
-load_PDB_components false
```

The benchmark antibodies are designed with the following commands (with variations for each protocol):

Command:

```
Antibody_designer.mpi.linuxgccrelease @common_flags.txt
@experimental_flags.txt –l PDBLIST.txt
```

Contents of ***common_flags.txt*** *file:*

```
-graft_design_cdrs L1 L2 L3 H1 H2
-seq_design_cdrs L1 L2 L3 H1 H2 H3

-output_ab_scheme AHo_Scheme
-nstruct 100
-outer_cycle_rounds 100
-random_start True
-add_graft_log_to_pdb
-ignore_zero_occupancy false
-ignore_unrecognized_res
-pdb_comments
```

```
-ex1
-ex2
-use_input_sc
-out:pdb_gz
-other_pose_to_scorefile
-scorefile_format json
-flip_HNQ
-delete_old_poses
-load_PDB_components false
```

*Contents of **experimental_flags.txt** file (in separate files for each example listed below as separated by the # sign):*

```
#Lambda/opt-E
      -light_chain lambda

#Lambda/opt-E/No antigen
      -light_chain lambda
      -remove_antigen True

#Lambda/opt-dG
      -light_chain lambda
      - mc_optimize_dG

#Lambda/opt-dG/No antigen
      -light_chain lambda
      -mc_optimize_dG
      -remove_antigen True

#Kappa/opt-E
      -light_chain kappa

#Kappa/opt-E/No antigen
      -light_chain kappa
      -remove_antigen True

#Kappa/opt-dG
      -light_chain kappa
      - mc_optimize_dG

#Kappa/opt-dG/No antigen
      -light_chain kappa
      - mc_optimize_dG
      -remove_antigen True
```

Contents of CDR Instruction File:

```
#RAbD Defaults (Used always, unless command override):
      L1 MinProtocol Min_Neighbors L2 L3
      L2 MinProtocol Min_Neighbors L1
      L3 MinProtocol Min_Neighbors L1 H3
      H1 MinProtocol Min_Neighbors H2 H3
      H2 MinProtocol Min_Neighbors H1
      H3 MinProtocol Min_Neighbors L1 L3
      ALL MinProtocol MinType min
```

```
#Experiments
	ALL CDRSet CLUSTER_CUTOFFS 5
```

For the redesign of the 2j88 antibody, the Rosetta command and files were:

Command:

```
antibody_designer.mpi.linuxgccrelease @common_flags.txt
@experimental_flags.txt –cdr_instructions cdr_instructions.txt –
s 2j88_pareto_optimal.pdb –nstruct 1000
```

Contents of ***common_flags.txt*** *file:*

```
-output_ab_scheme AHo_Scheme
-add_graft_log_to_pdb
-ignore_zero_occupancy false
-ignore_unrecognized_res
-pdb_comments
-ex1
-ex2
-use_input_sc
-out:pdb_gz
-other_pose_to_scorefile
-scorefile_format json
-flip_HNQ
-delete_old_poses
-load_PDB_components false
```

Contents of ***experimental_flags.txt*** *file, Docking Off:*

```
-outer_cycle_rounds 100
-s input_pdbs/pareto_2j88_renum_0002.pdb.gz
-run_relax
-random_start
-design_protocol even_cluster_mc
-light_chain kappa
```

Contents of ***experimental_flags.txt****, Docking On:*

```
-outer_cycle_rounds 100
-run_relax
-random_start
-design_protocol even_cluster_mc
-light_chain kappa
-use_epitope_constraints
-do_dock
-inner_cycle_rounds 2
```

Contents of ***cdr_instructions.txt****, H2 Design:*

```
H2 ALLOW
ALL GraftDesign mintype relax
H2 MinProtocol Min_Neighbors H1 H3
H2 CDRSet Cluster_Cutoffs 10
ALL CDRSET EXCLUDE PDBIDs 2J88
```

Contents of ***cdr_instructions.txt****, L1 Design:*

```
L1 ALLOW
ALL GraftDesign mintype relax
L1 GraftDesign Min_Neighbors L3 L4
```

```
L1 CDRSet Cluster_Cutoffs 10
ALL CDRSET EXCLUDE PDBIDs 2J88
```

Contents of ***cdr_instructions.txt****, L1/L4 Design:*

```
L1 ALLOW
ALL GraftDesign mintype relax
L1 GraftDesign Min_Neighbors L3 L4
L1 CDRSet Cluster_Cutoffs 10
ALL CDRSET EXCLUDE PDBIDs 2J88
L4 SeqDesign ALLOW
```

Note that many options, such as which CDRs to design, can alternatively be set via simple command-line options.

For the redesign of the 4JAN antibody, the Rosetta command and files were:

General Command:

```
antibody_designer.mpi.linuxgccrelease @common_flags.txt –
cdr_instructions cdr_instructions.txt –s 4JAN_pareto_optimal.pdb
–nstruct 250
```

Contents of ***common_flags.txt*** *file:*

```
-outer_cycle_rounds 200
-run_relax
-random_start
-design_protocol even_cluster_mc
-light_chain lambda
-add_graft_log_to_pdb
-ignore_zero_occupancy false
-ignore_unrecognized_res
-pdb_comments
-ex1
-ex2
-use_input_sc
-out:pdb_gz
-other_pose_to_scorefile
-scorefile_format json
-flip_HNQ
-delete_old_poses
-load_PDB_components false
```

Contents of ***cdr_instructions.txt****, H2 Design, only clusters with profiles:*

```
H2 ALLOW
H2 MinProtocol Min_Neighbors H1 H3
H2 CDRSet Cluster_Cutoffs 10
ALL CDRSet Exclude PDBIDs 4JAN
```

Contents of ***cdr_instructions.txt****, H2 Design, all:*

```
H2 ALLOW
H2 MinProtocol Min_Neighbors H1 H3
ALL CDRSet Exclude PDBIDs 4JAN
```

Contents of ***cdr_instructions.txt****, L1/L3 Design, only clusters with profiles:*

```
L1 ALLOW
L3 ALLOW
L3 MinProtocol Min_Neighbors L1 H3
L1 MinProtocol Min_Neighbors L3 H3
```

```
L1 CDRSet Cluster_Cutoffs 10
L3 CDRSet Cluster_Cutoffs 10
ALL CDRSet Exclude PDBIDs 4JAN
```

*Contents of **cdr_instructions.txt**, L1/L3 Design, all:*

```
L1 ALLOW
L3 ALLOW
L3 MinProtocol Min_Neighbors L1 H3
L1 MinProtocol Min_Neighbors L3 H3
ALL CDRSet Exclude PDBIDs 4JAN
```

The command for the AntibodyFeature reporters (described below) used in the analysis of the antibodies was:

Command:

```
rosetta_scripts.macosxrelease –l DECOYS.txt @common_flags –
parser:protocol features_script.xml
```

Contents of features_script.xml:

```
<ROSETTASCRIPTS>
   <MOVERS>
      <ReportToDB name='features' database_name='my_db'>
         <feature name='CDRClusterFeatures' numbering_scheme='AHO_Scheme'/>
         <feature name='AntibodyFeatures' numbering_scheme='AHO_Scheme'
cdr_definition='North' interface='LH_A' pack_separated='1' pack_together='1'/>
         <feature name='ResidueFeatures'/>
         <feature name='PdbDataFeatures'/>
         <feature name='ResidueTypesFeatures'/>
         <feature name='ScoreTypeFeatures'/>
         <feature name='StructureScoresFeatures'/>
         <feature name='ResidueSecondaryStructureFeatures'/>
         <feature name='ResidueScoresFeatures'/>
         <feature name='ResidueBurialFeatures'/>
      </ReportToDB>
   </MOVERS>
   <PROTOCOLS>
      <Add mover_name='features'/>
   </PROTOCOLS>
</ROSETTASCRIPTS>
```

Once each database was created, extra output in each decoy PDB file (added through the option *-add_graft_log_to_pdb),* was used to calculate recoveries and risk ratios through the creation and use the Bio-Jade AnalyzeRecovery module of the RAbD_BM subpackage. (https://bio-jade.readthedocs.io/en/latest/).

2. Dihedral, Epitope, and Paratope Constraints

Several constraint types are used by the Antibody Design framework to limit unproductive structural perturbations of the CDR regions and the relative orientation of the antibody-antigen interface while docking in the program. There are many constraint types with associated function types implemented in Rosetta. These constraints are evaluated via terms added to the Rosetta energy function. The Rosetta energy minimizer (which optimizes the conformation of the structure by finding the local energy minimum) can use these constraints to find optimal values

that help to satisfy all the energy terms including the constraints. Within the Rosetta Antibody Design framework, the weight of these constraints can be set from command-line options. We can set parameters that govern whether these constraints are used throughout the protocol (where they also act as structural filters) or only in certain situations like minimization or docking (where they act only to guide the structure to an optimum conformation that satisfies the constraints).

The set of general Antibody constraint movers that were implemented consist of the *CDRDihedralConstraintMover,* *ParatopeSiteConstraintMover,* and the *ParatopeEpitopeSiteConstraintMover.* These Movers (a mover applies some change to a Pose or structure [1]) can be fine-tuned for specific design strategies using a number of user-accessible options and RosettaScripts [2].

The *CDRDihedralConstraintMover* places Circular Harmonic constraints on each ϕ and ψ dihedral angle of a given CDR as cluster-specific and general-use constraints. The equation for the Circular Harmonic constraint is as follows where x_0 is the starting dihedral angle, x is the changed dihedral angle, and σ is the standard deviation of x:

$$f(x) = \left(\frac{NearestAngleRadians(x, x_0) - x_0}{\sigma} \right)^2$$

Constraints are added to help Rosetta maintain a particular loop structure during any backbone optimization. Dihedral constraints are used instead of coordinate constraints (which try to keep each atom at a particular Cartesian coordinate) in order to allow more natural, hinge-like motion of the CDR loops. Users of the protocol who wish to design antibodies without these constraints can set the weight of the dihedral constraint to zero via a command-line option.

The cluster-specific constraints have the value of x_0 and standard deviation for each backbone ϕ and ψ dihedral angle at the angle mean and standard deviation of the members of the cluster. These constraints are output by PyIgClassify using a high-quality set of non-redundant data. The default behavior of the *CDRDihedralConstraintMover* is to add these constraints for a particular CDR only if there are enough members in the cluster to have reliable data. If data are scarce, then general dihedral constraints are added, with means at the current angles and a standard deviation that was originally compiled by taking the mean of the standard deviations of all CDR clusters. These angles can be set via command-line options. By default, we use these general dihedral constraints for H3, since it does not cluster well.

For the cluster-specific constraints (and other places in the protocol), we generally filter out outliers in the data as described below. This can be turned off through the use of an option that will load a different set of constraints compiled with structures that are not filtered for outliers. This can be useful if using outliers elsewhere in the protocol.

SiteConstraints are a set of atom-pair constraints that evaluate whether a residue interacts with some other chain or region -- roughly, that it is (or is not) in a binding site. More specifically, if we have a *SiteConstraint* on a particular residue, that *SiteConstraint* consists of a set of distance constraints on the Cα atom from that residue to the Cα atom of all other residues in a set, typically the set being specific residues on another chain or chains. After each constraint is evaluated, *only* the constraint giving the lowest score is used as the *SiteConstraint* energy for that residue. These *SiteConstraints* use a Flat Harmonic function by default:

$$\begin{aligned} f(x) &= k(x - x_0)^2 \qquad |x - x_0| > T \\ &= 0 \qquad\qquad\quad\;\; |x - x_0| \le T \end{aligned}$$

Values of the standard deviation are set at 1 Å, while the tolerance is set at the interface distance of the protocol (8 Å default), which means that there is no penalty for the *SiteConstraint* except at distances greater than this distance.

The *ParatopeSiteConstraintMover* adds *SiteConstraints* between each CDR residue and the antigen. This helps to keep the CDR paratope at the interface during docking; without it, docking can use the whole of the antibody surface instead of just the paratope and this can be seen in resulting models. These paratope constraints are added automatically in the program, and the CDRs of the paratope can be controlled through an option.

The *ParatopeEpitopeSiteConstraintMover* adds *SiteConstraints* from the epitope residues to the paratope residues and from the paratope to the epitope. Target epitope residues can be specified via command-line or automatically detected via the set interface distance. These constraints are off by default, but if they are enabled, they are set instead of the *ParatopeSiteConstraintMover* and help to keep the paratope and the epitope in contact during design when the docking component of the algorithm is enabled.

3. Outlier Control

In our original clustering of the antibody CDR structures, an affinity propagation clustering technique was used on a carefully curated dataset of high-resolution structures and few outliers [3]. In order to match new CDR structures with a proper cluster from that original clustering, we use the dihedral angle metric originally used for the affinity propagation, but measure it against the centroid (representative structure) of all clusters of the same length. The cluster with the lowest dihedral distance is assigned as the cluster for that structure [4].

While this is useful to assign CDRs of known length to a particular cluster, many structures become outliers of the particular cluster and would have formed their own cluster if clustering was repeated (Kelow and Dunbrack, in preparation). To optimize our CDR profiles, constraints, and other aspects of the design program for an updated database, we needed to quantitatively define what would be considered an outlier.

We used both the dihedral distance metric and RMSD of all backbone atoms to help define an outlier. In order to visualize the breadth of each cluster, we generated PyMol sessions of each of the clusters using python and PyRosetta [5] by aligning the CDRs to their cluster center either using all backbone heavy atoms or by aligning only the stem region (three framework residues on either side of the CDR loop). We also generated plots of dihedral distance versus RMSD and length versus RMSD for both alignment types and for each length and cluster, where high RMSD can be seen even with lower dihedral distance, especially when only the stem was aligned. We then used these plots and the PyMol visualizations for each CDR cluster to define two outlier definitions – one conservative and one liberal (used as the default). We calculate the RMSD for these definitions through the full CDR alignments as the stem alignment can result in very high RMSD for low dihedral angle distances, attributable to hinge-like motions in the CDR:

(Conservative):

```
if (DihDis ≥ 40° OR RMSD ≥ 1.5 Å) then CDR is Outlier
```

(Liberal):

```
if (DihDis ≥ 40° AND RMSD ≥ 1.5 Å) then CDR is Outlier
```

Outlier control is handled as an option in the Antibody Design framework, where each set of data used by the framework is first generated with and without outliers and using both the

liberal and conservative definition of an outlier. For smaller clusters or H3 (which only cluster well at lengths ≤ 9), outliers may be useful in the design search and an option will switch all aspects of the framework to include outliers for sequence and dihedral constraint statistics as well as graft sets. By default, outliers are left out, but used for H3 since it does not cluster well.

4. Antibody Feature Analysis

Three FeatureReporters were developed as a part of the Rosetta Feature Reporter framework [6-8] to aid in the modeling and design of antibody structures. Each of these can be used through the RosettaScript framework on a list of structures. The physical attributes reported are output to a relational database, such as SQLITE3, across multiple tables for further analysis. These databases can easily be converted into CSV files or read by available packages in R and Python.

The *CDRClusterFeature* reporter identifies all North/Dunbrack CDR clusters in an antibody to the closest cluster centroid using the same metric described in PyIgClassify as well as information pertaining to the dihedral distances of the CDRs [4]. It is the primary FeatureReporter used in benchmarking length and cluster recovery**.** The database tables output by the *CDRClusterFeatures* are detailed in Table D in S1 Supporting Information.

The *InterfaceFeature Reporter,* detailed in Table E in S1 Supporting Information, analyzes protein-protein and protein-ligand interfaces, outputting a number of different tables and physical data. Much of the analysis is done through the Rosetta InterfaceAnalyzer [9,10] which we have updated. The InterfaceAnalyzer calculates differences in scoring (such as an estimate of the interface **Δ**G – the enthalpic component of the full binding free energy) by physically separating the interface components (such as antibody from antigen) and optimizing interface residue side chains - both in the complexed and separated conformations. An interface distance of 6 Å is used as the default interface distance.

Separate tables are output for the overall complex, the individual proteins in the complex, and the interface residues. The main data output by this Reporter are the estimated binding energy (**Δ**G) of the complex in Rosetta Energy Units (REU), the change in solvent accessible surface area upon binding (**Δ**SASA) using the Le Grand SASA calculation method [11], the Lawrence and Colman shape complementary of the interface (sc_value) [12], the packing quality (packstat) [13], and the number of unsaturated hydrogen bonds in the complex [10].

We added alternative SASA radius sets to Rosetta, with the standard, now-defunct radii changing from the default to 'legacy'. We implemented a variety of radius sets found in the literature and used in various structural modeling programs in which they either implicitly or explicitly include hydrogen atoms. Once a particular radius set is used, the SASA machinery will change its consideration of implicitly or explicitly including hydrogen atoms during the calculation depending on the set.

The atomic radius set with implicit hydrogens is the one used by the program Naccess, a popular program used for the calculation of SASA [14]. This set was derived by Chothia in his seminal 1976 paper [15], while explicit hydrogen radius sets include the legacy radii, the Rosetta Lennard-Jones (LJ) radii (which are mostly the same as the LJ radii from the CHARMM molecular dynamics program [16]), and the radii used by the program *reduce* (a program for the placement of hydrogens onto molecular models and crystal structures) [17], originating from physical data obtained from Bondi [18] and Gavezzotti [19]. The reduce radius set is now the default in Rosetta.

We implemented the *AntibodyFeature* Reporter, a type of *InterfaceFeature* Reporter specific for antibody and antibody-antigen interfaces, while outputting a number of *additional* metrics for antibodies and CDRs. Some of the main metrics include CDR, antibody, and paratope charge, ΔG and ΔSASA, H3 kink statistics, number of contacts, and packing angle statistics [20].

The packing angle is a measure of the relative orientation between the light and heavy antibody chains. It uses four conserved residues of each chain in the framework beta-sheets at the VL and VH interface and principal component analysis to define four centroid points and a dihedral angle for which to quantify the orientation [21].

A full list of the metrics and tables output by the *AntibodyFeature* Reporter can be found in Table F in S1 Supporting Information. All tables output by the *InterfaceFeature* Reporter are output by the *AntibodyFeature* Reporter for specific antibody interfaces specified where A is the antigen: LH-A, L-H, L-A, H-A.

REFERENCES

1. Leaver-Fay A, Tyka M, Lewis SM, Lange OF, Thompson J, Jacak R, Kaufman K, Renfrew PD, Smith CA, Sheffler W, Davis IW, Cooper S, Treuille A, Mandell DJ, Richter F, Ban YE, Fleishman SJ, Corn JE, Kim DE, Lyskov S, Berrondo M, Mentzer S, Popovic Z, Havranek JJ, Karanicolas J, Das R, Meiler J, Kortemme T, Gray JJ, Kuhlman B, Baker D, Bradley P (2011). Rosetta3 an object-oriented software suite for the simulation and design of macromolecules. *Methods Enzymol* **487**: 545-574.
2. Fleishman SJ, Leaver-Fay A, Corn JE, Strauch EM, Khare SD, Koga N, Ashworth J, Murphy P, Richter F, Lemmon G, Meiler J, Baker D (2011). RosettaScripts: a scripting language interface to the Rosetta macromolecular modeling suite. *PLOS ONE* **6**: e20161.
3. North B, Lehmann A, Dunbrack RL, Jr. (2011). A new clustering of antibody CDR loop conformations. *J Mol Biol* **406**: 228-256.
4. Adolf-Bryfogle J, Xu Q, North B, Lehmann A, Dunbrack RL, Jr. (2015). PyIgClassify: a database of antibody CDR structural classifications. *Nucleic Acids Res* **43**: D432-438.
5. Chaudhury S, Lyskov S, Gray JJ (2010). PyRosetta: a script-based interface for implementing molecular modeling algorithms using Rosetta. *Bioinformatics* **26**: 689-691.
6. Leaver-Fay A, O'Meara MJ, Tyka M, Jacak R, Song Y, Kellogg EH, Thompson J, Davis IW, Pache RA, Lyskov S, Gray JJ, Kortemme T, Richardson JS, Havranek JJ, Snoeyink J, Baker D, Kuhlman B (2013). Scientific benchmarks for guiding macromolecular energy function improvement. *Methods Enzymol* **523**: 109-143.
7. Ihaka R, Gentleman R (1996). R: A language for data analysis and graphics. *J Comp Graphical Stat* **5**: 299-314.
8. R Core Team (2015) R: A Language and Environment for Statistical Computing. Vienna, Austria: R Foundation for Statistical Computing.
9. Lewis SM, Kuhlman BA (2011). Anchored design of protein-protein interfaces. *PLOS ONE* **6**: e20872.
10. Stranges PB, Kuhlman B (2013). A comparison of successful and failed protein interface designs highlights the challenges of designing buried hydrogen bonds. *Protein Sci* **22**: 74-82.
11. Le Grand SM, Merz KM (1993). Rapid approximation to molecular surface area via the use of Boolean logic and look - up tables. *J Comput Chem* **14**: 349-352.
12. Lawrence MC, Colman PM (1993). Shape complementarity at protein/protein interfaces. *J Mol Biol* **234**: 946-950.
13. Sheffler W, Baker D (2009). RosettaHoles: rapid assessment of protein core packing for structure prediction, refinement, design, and validation. *Protein Sci* **18**: 229-239.
14. Hubbard SJ, Thornton JM (1993) NACCESS. London: Department of Biochemistry and Molecular Biology, University College London.

15. Chothia C (1976). The nature of the accessible and buried surfaces in proteins. *J Mol Biol* **105**: 1-12.
16. MacKerell AD, Bashford D, Bellott M, Dunbrack RL, Evanseck JD, Field MJ, Fischer S, Gao J, Guo H, Ha S, Joseph-McCarthy D, Kuchnir L, Kuczera K, Lau FT, Mattos C, Michnick S, Ngo T, Nguyen DT, Prodhom B, Reiher WE, Roux B, Schlenkrich M, Smith JC, Stote R, Straub J, Watanabe M, Wiorkiewicz-Kuczera J, Yin D, Karplus M (1998). All-atom empirical potential for molecular modeling and dynamics studies of proteins. *J Phys Chem B* **102**: 3586-3616.
17. Word JM, Lovell SC, LaBean TH, Taylor HC, Zalis ME, Presley BK, Richardson JS, Richardson DC (1999). Visualizing and quantifying molecular goodness-of-fit: small-probe contact dots with explicit hydrogen atoms. *J Mol Biol* **285**: 1711-1733.
18. Bondi A (1964). van der Waals volumes and radii. *The journal of physical chemistry* **68**: 441-451.
19. Gavezzotti A (1983). The calculation of molecular volumes and the use of volume analysis in the investigation of structured media and of solid-state organic reactivity. *J Am Chem Soc* **105**: 5220-5225.
20. Abhinandan KR, Martin AC (2010). Analysis and prediction of VH/VL packing in antibodies. *Protein Eng Des Sel* **23**: 689-697.
21. Marze NA, Lyskov S, Gray JJ (2016). Improved prediction of antibody VL-VH orientation. *Protein Eng Des Sel* **29**: 409-418.
