# Supplementary material for: RosettaAntibodyDesign (RAbD): A general framework for computational antibody design
Source: PLoS Comput Biol. 2018 Apr 27;14(4):e1006112. doi: 10.1371/journal.pcbi.1006112 (PMC5942852; doi:10.1371/journal.pcbi.1006112)
Supplement: S1 Supporting Information — (PDF) [file pcbi.1006112.s002.pdf]

S1 Supporting Information
Adolf-Bryfogle et al., RosettaAntibodyDesign: A general and flexible framework for computational antibody design

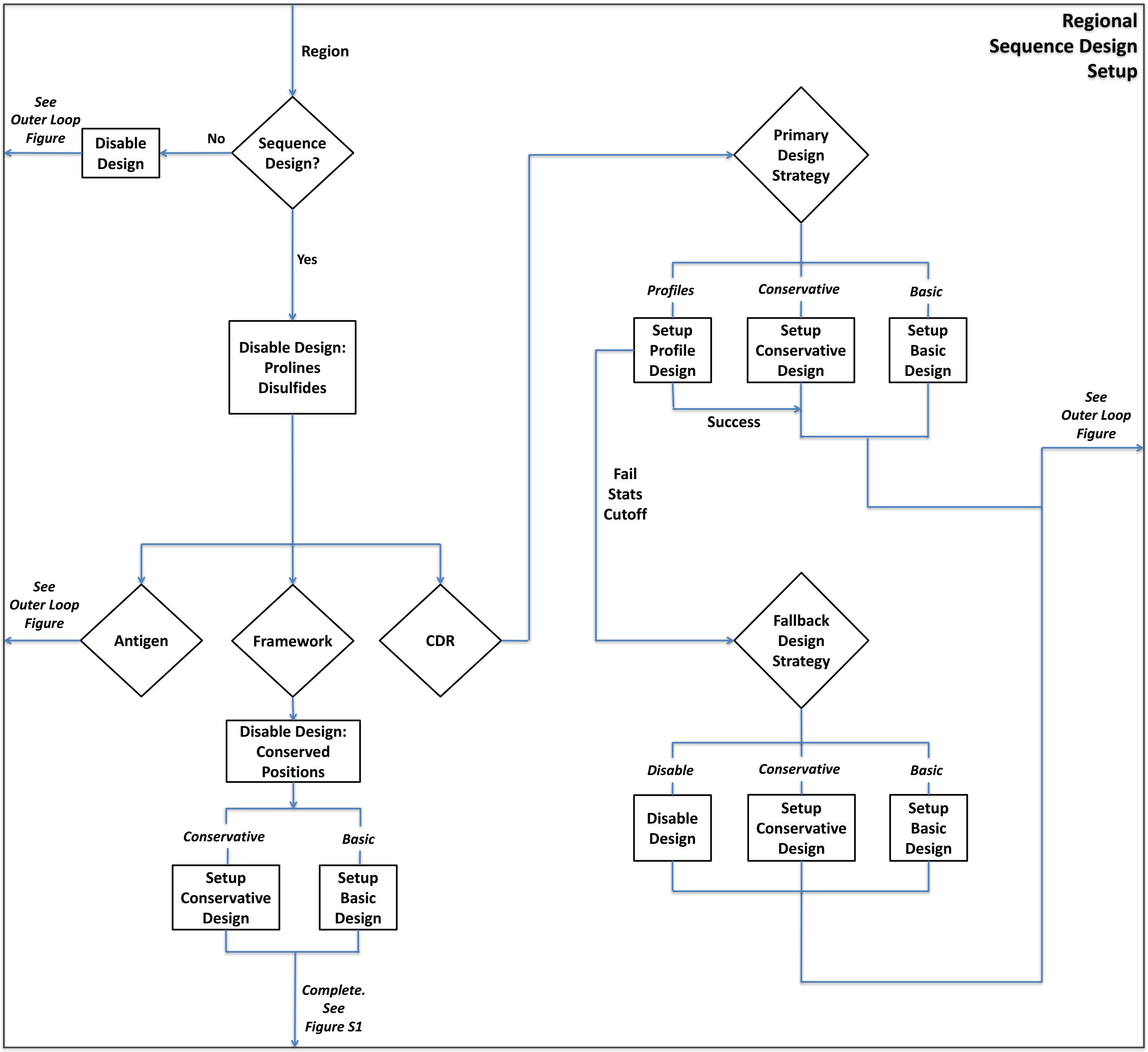

Fig. A. Regional sequence design setup. For each region (Antigen, Framework, Each CDR), whether that region is set to design is determined from the program options and CDR Instructions File. If the region is set to design, design is disabled for prolines and disulfide-bonded cysteine residues within the region by default. If it is an antigen region, we use "basic design" (standard Rosetta design) and exit the setup. By default, the framework region is held fixed in sequence space. But if framework design is enabled, we disable design on completely conserved positions, such as the tryptophan immediately after the H3 loop, and perform conservative or basic design on the rest. Finally, when designing a CDR region, we set up the Primary Sequence Design strategy that is set by the CDR Instructions File. If the Primary Sequence Design strategy is to use the CDR cluster-based profiles and there is scarce data, we use the set Fallback Design Strategy. After the CDR Sequence Design strategy is done, we have completed the setup for sequence design.

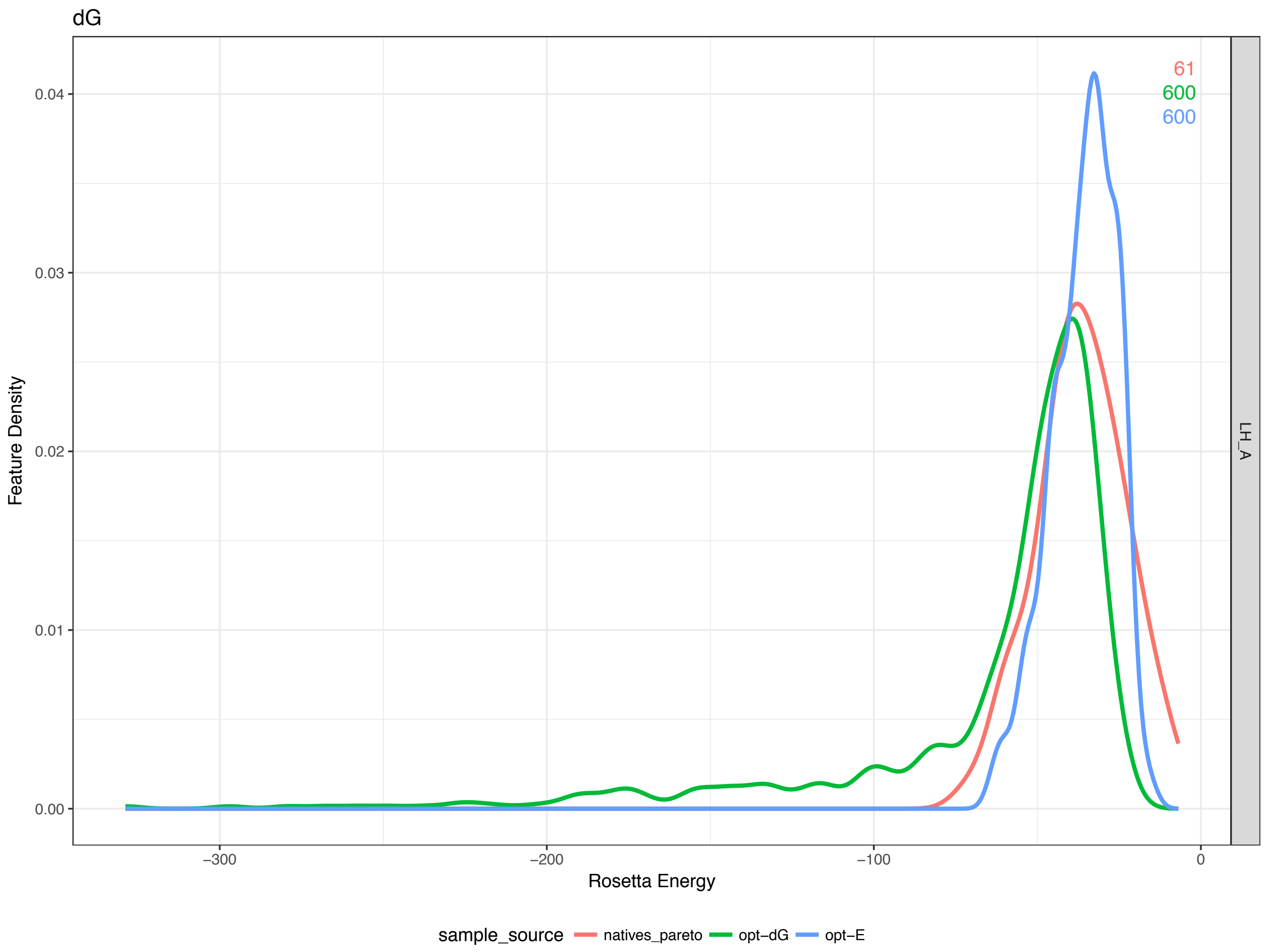

Fig. B. Benchmark Rosetta interface energies. Kernel density estimates (KDE) and averages of the Rosetta Interface Energy (dG) of the top 10% of the 100 decoys for each of the 60 antibody-antigen complexes in the opt-E and opt-dG benchmarks as well as the natives after optimization. As expected, opt-dG produces lower dG scores that are more similar to native than opt-E.

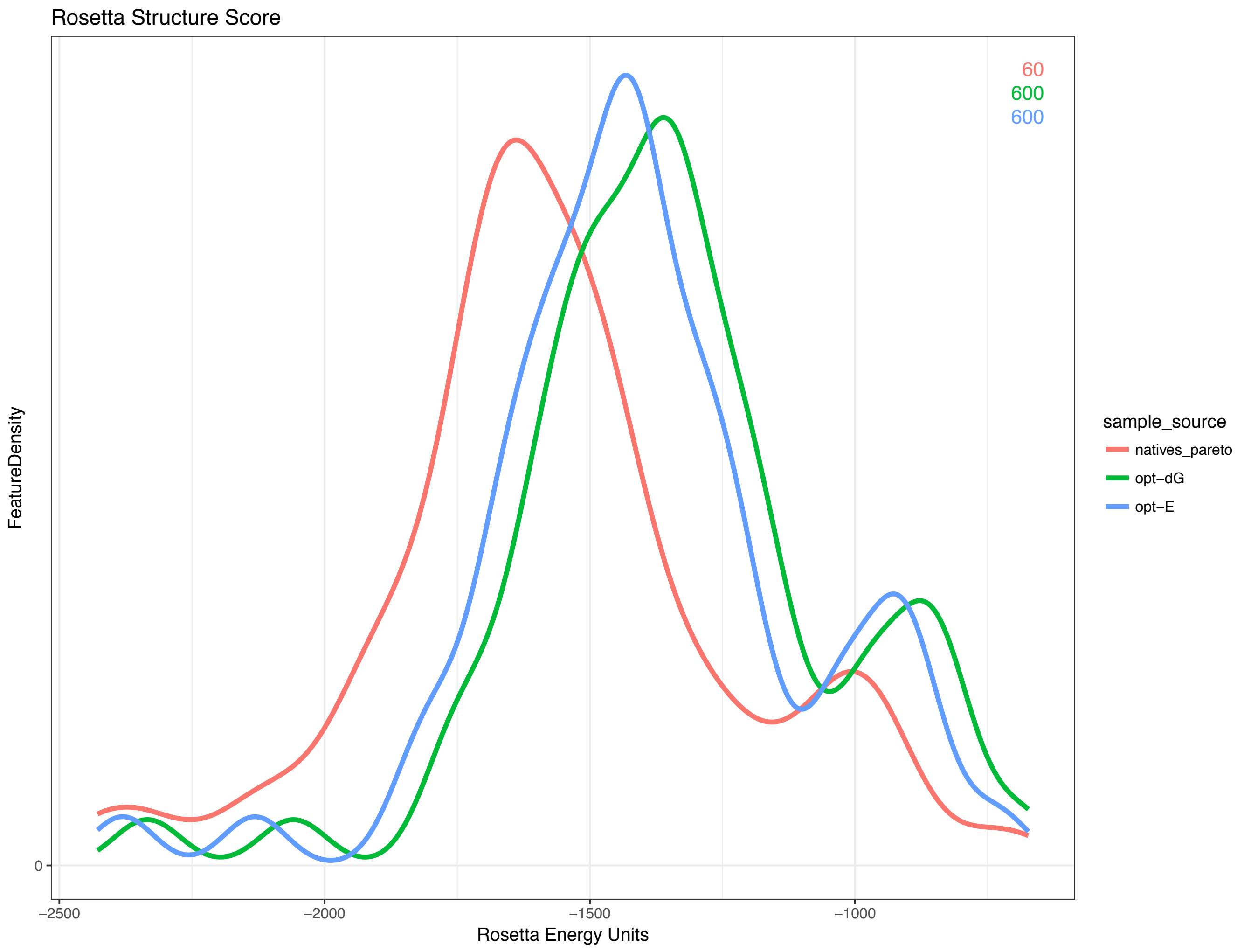

Fig. C. Benchmark total Rosetta energies. Kernel density estimates of total Rosetta energy (REU) of the opt-E vs opt-dG benchmark decoy set using the current Rosetta Energy function (REF2015). Densities for the natives and the top 10% of decoys are shown.

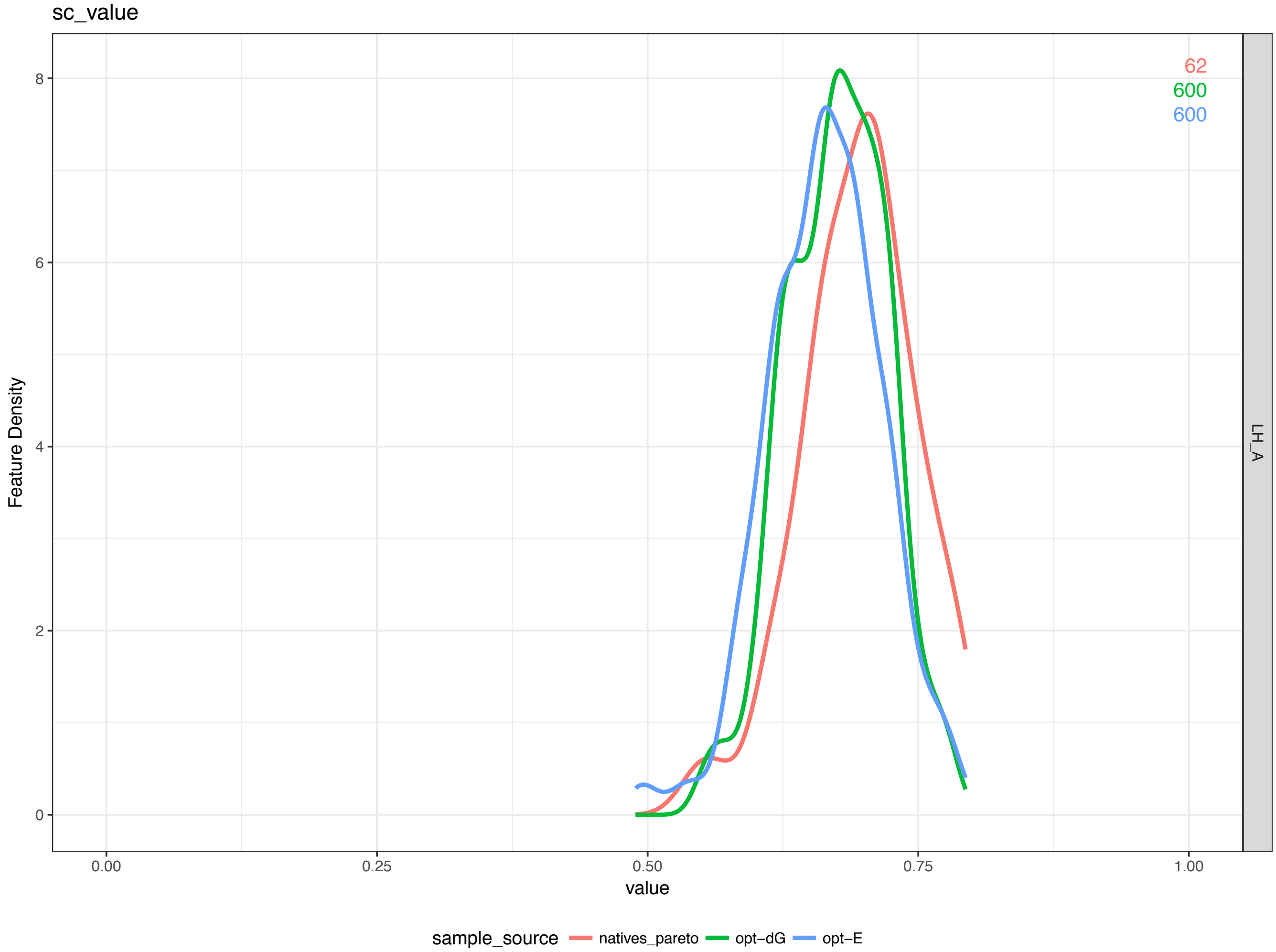

Fig. D. Benchmark shape complementarities. Kernel density estimates of the Lawrence and Colman Shape Complementarity value (*sc_value*) of the opt-E and opt-dG benchmark decoys for 60 antibody-antigen complexes using the Rosetta *AntibodyFeature* reporter.

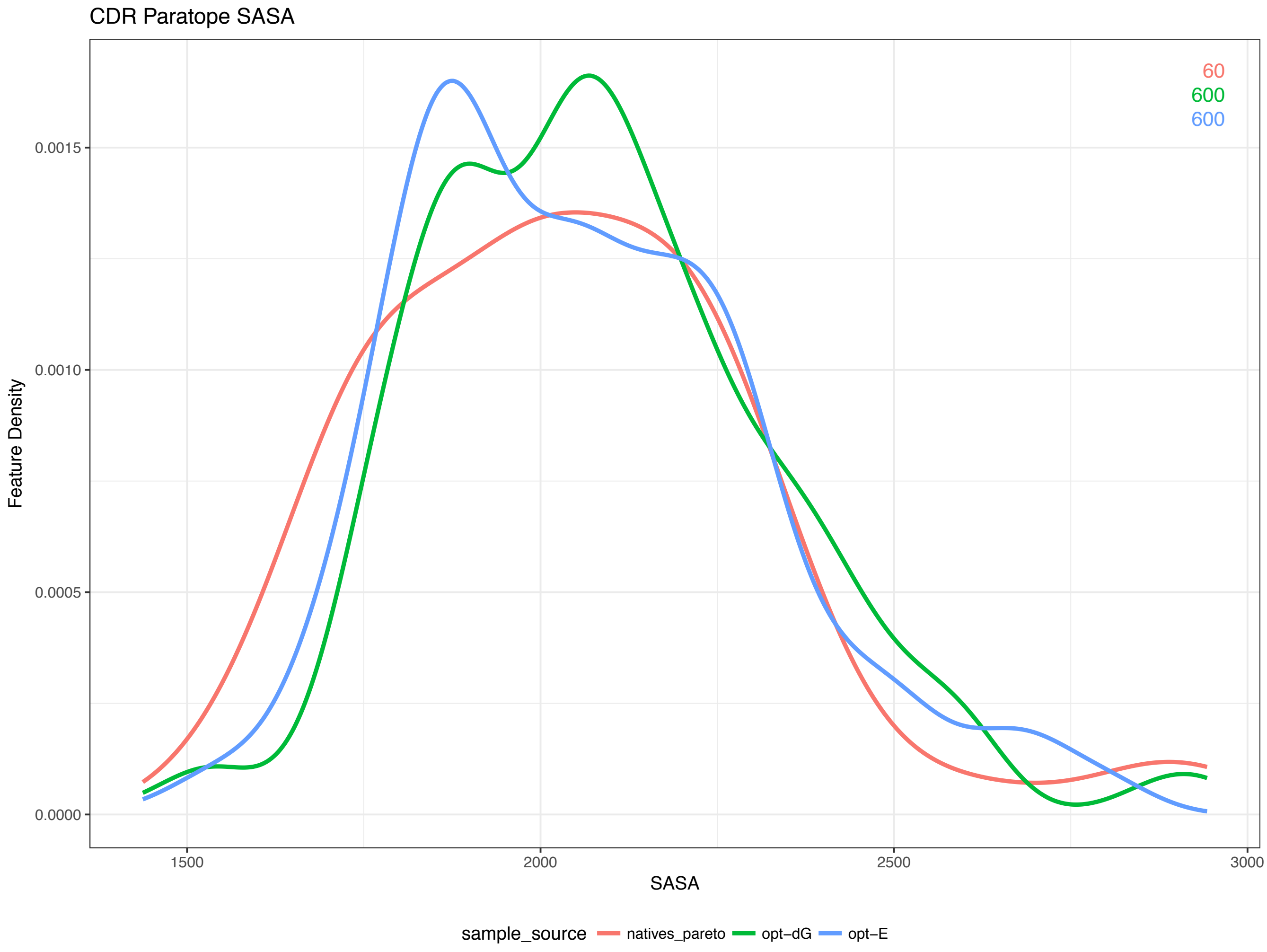

Fig. E. Benchmark solvent accessible surface areas. Kernel density estimates of the buried Solvent Accessible Surface Area (**Δ**SASA) of the opt-E and opt-dG 60 antibody benchmark decoy set at the antibody/antigen interface compared to the relaxed native structures.

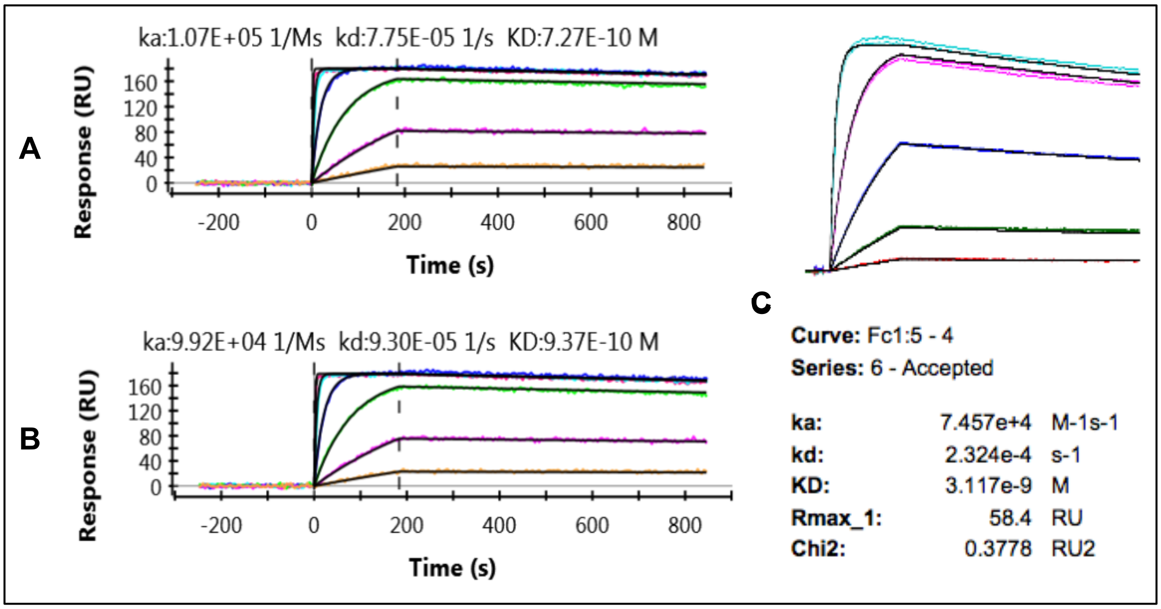

Fig. F. Kinetic sensorgrams of design L1_10 to Bee Hyaluronidase. (A) *XPR Repeat 1*; **(B)** *XPR Repeat 2*; **(C)** *Biacore 4000.*

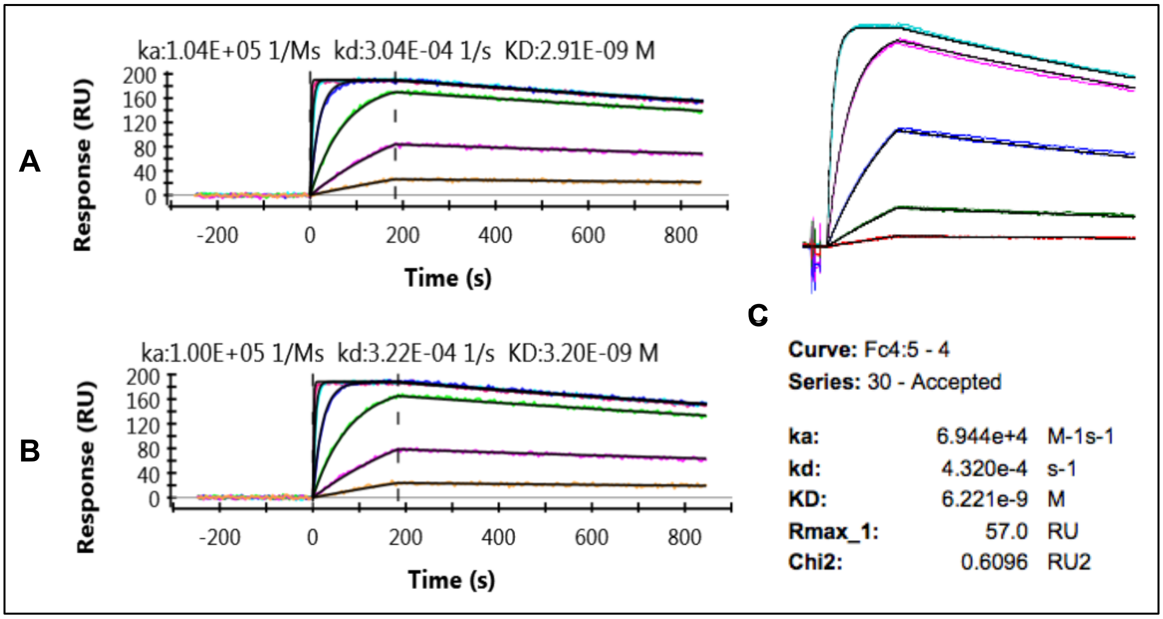

Fig. G. Kinetic sensorgrams of design L1_5 to Bee Hyaluronidase. (A) *XPR Repeat 1*; **(B)** *XPR Repeat 2*; **(C)** *Biacore 4000.*

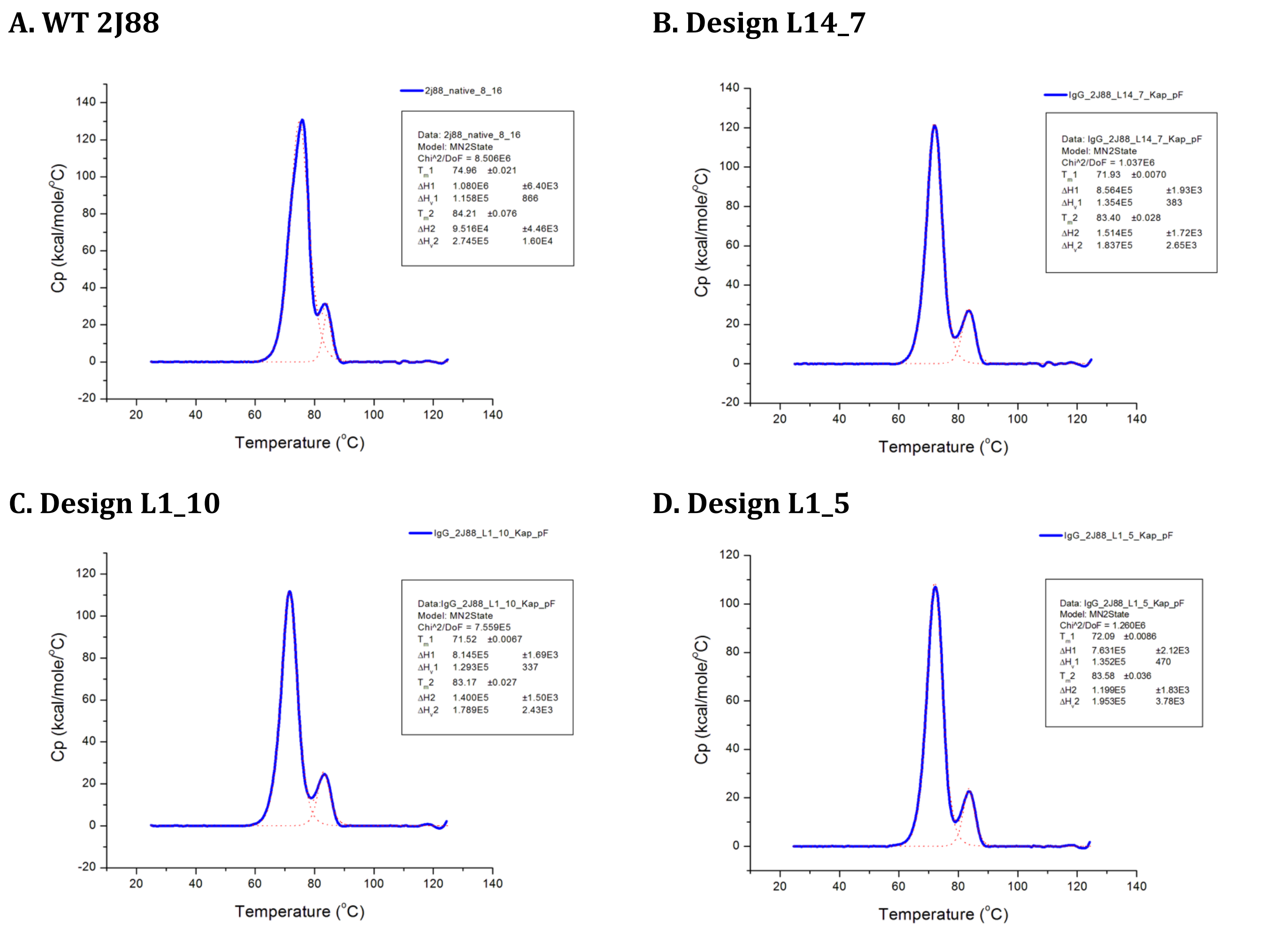

Fig. H. Thermostability measurements of WT 2J88 antibody and designs by Differential Scanning Calorimetry (DSC). (**A**) WT 2J88 thermostability; (**B**) L14_7 design thermostability; (**C**) L1_10 design thermostability; (**D**) L1_5 design thermostability.

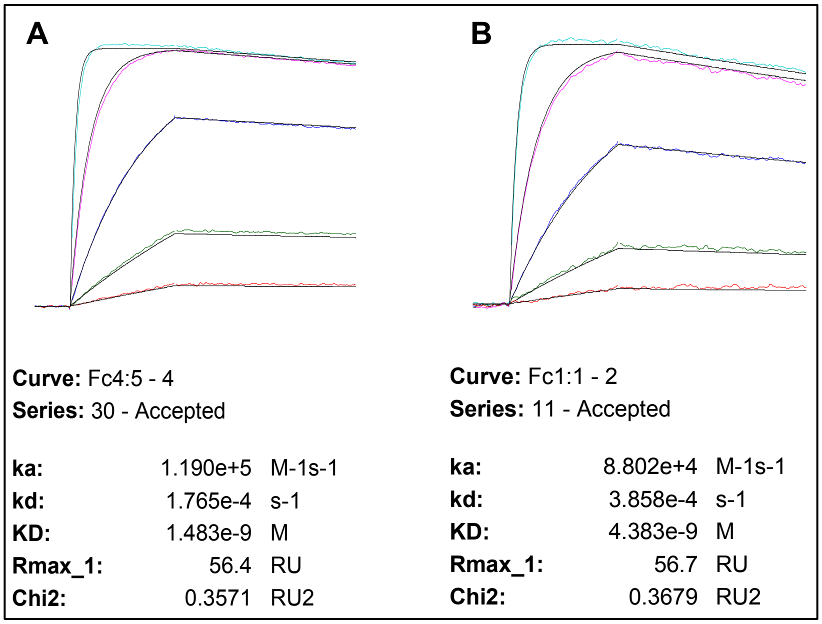

Fig. I. L14_7 Mutants with WT residue at position 38. (**A**) L14_7. (**B**) L14_7 K38Y.

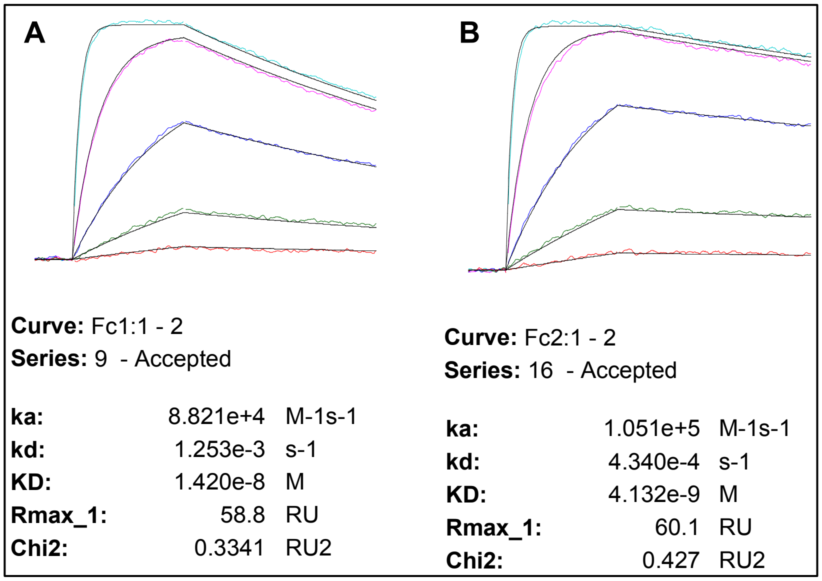

Fig. J. 2J88 WT Mutants with L14_7 residue at position 38. (A) 2J88 WT; **(B)** 2J88 Y38K

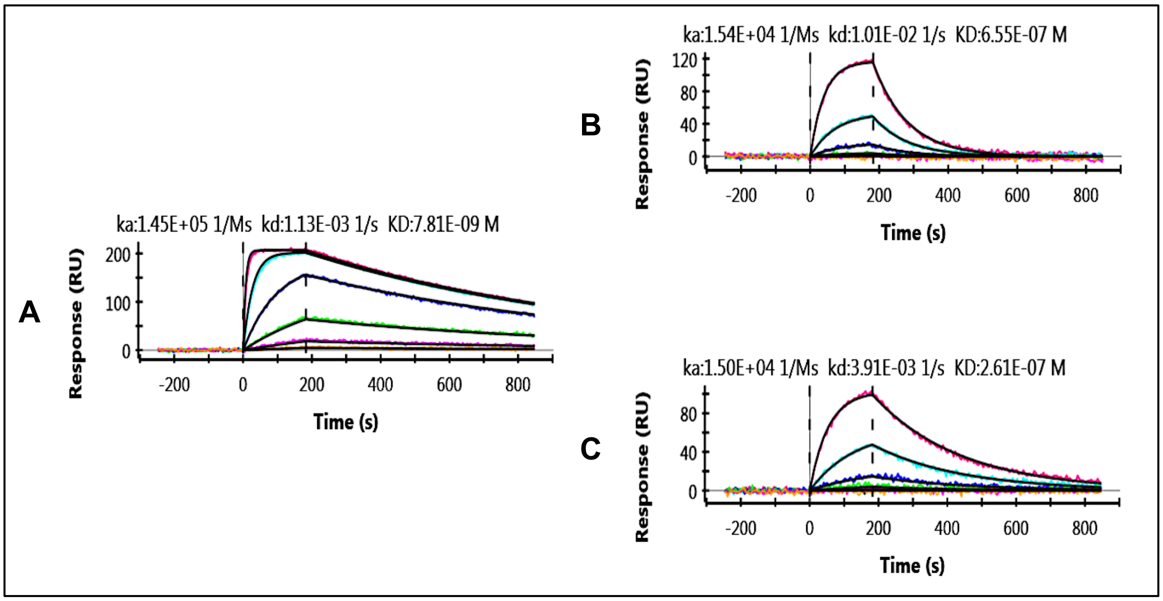

Fig. K. 2J88 WT Mutants with L14_7 residue at position 38. (A) 2J88 WT; **(B)** L1_4 Design; **(C)** L1_4 S36V.

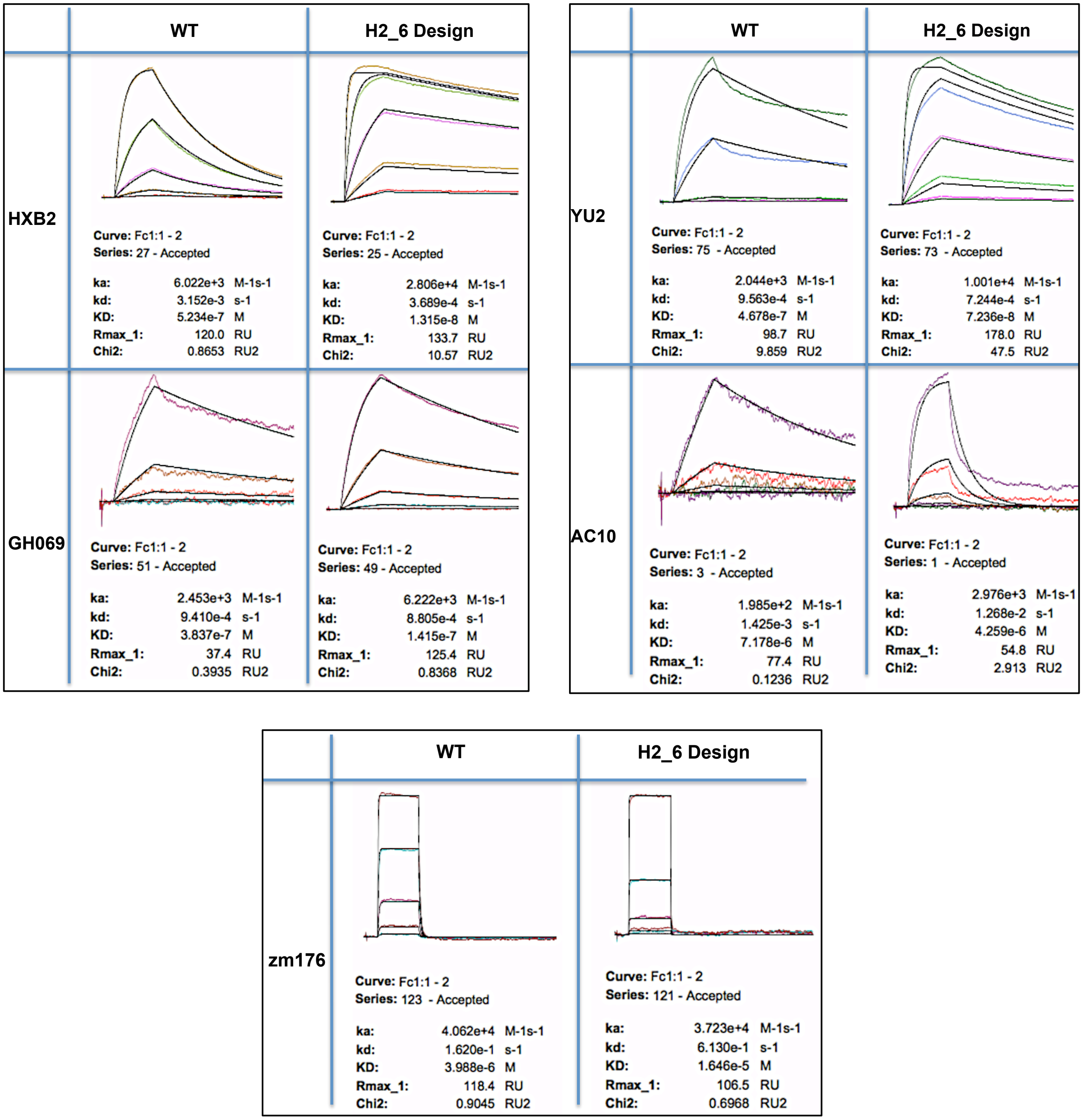

Fig. L. Kinetic sensorgrams of CH103 antibody and H2_6 design to a panel of gp120s. Binding studies were performed on a Biacore 4000.

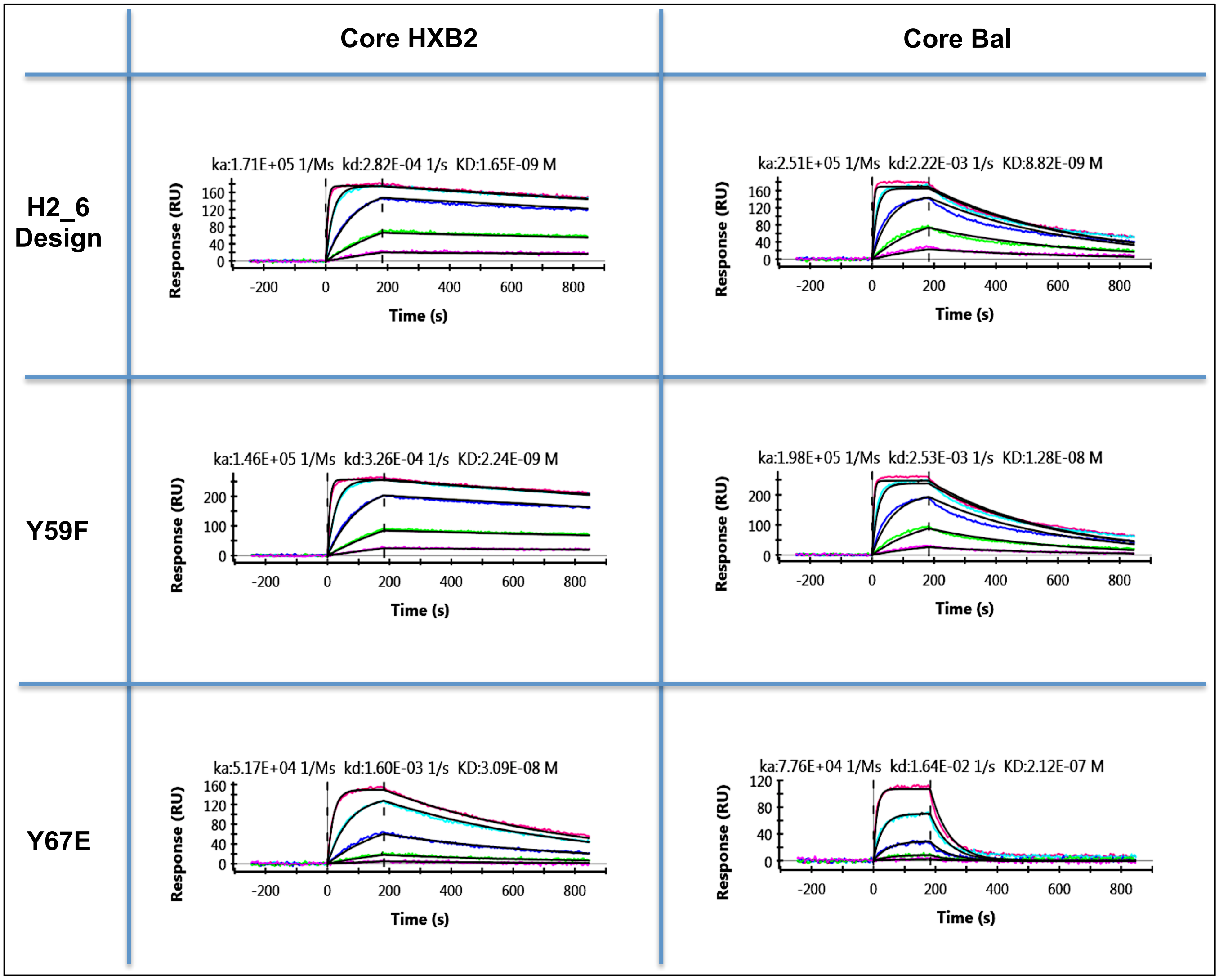

Fig. M. Kinetic Sensorgrams (ProteON XPR) of CH103, H2_6 design mutants with residues from CH103 WT.

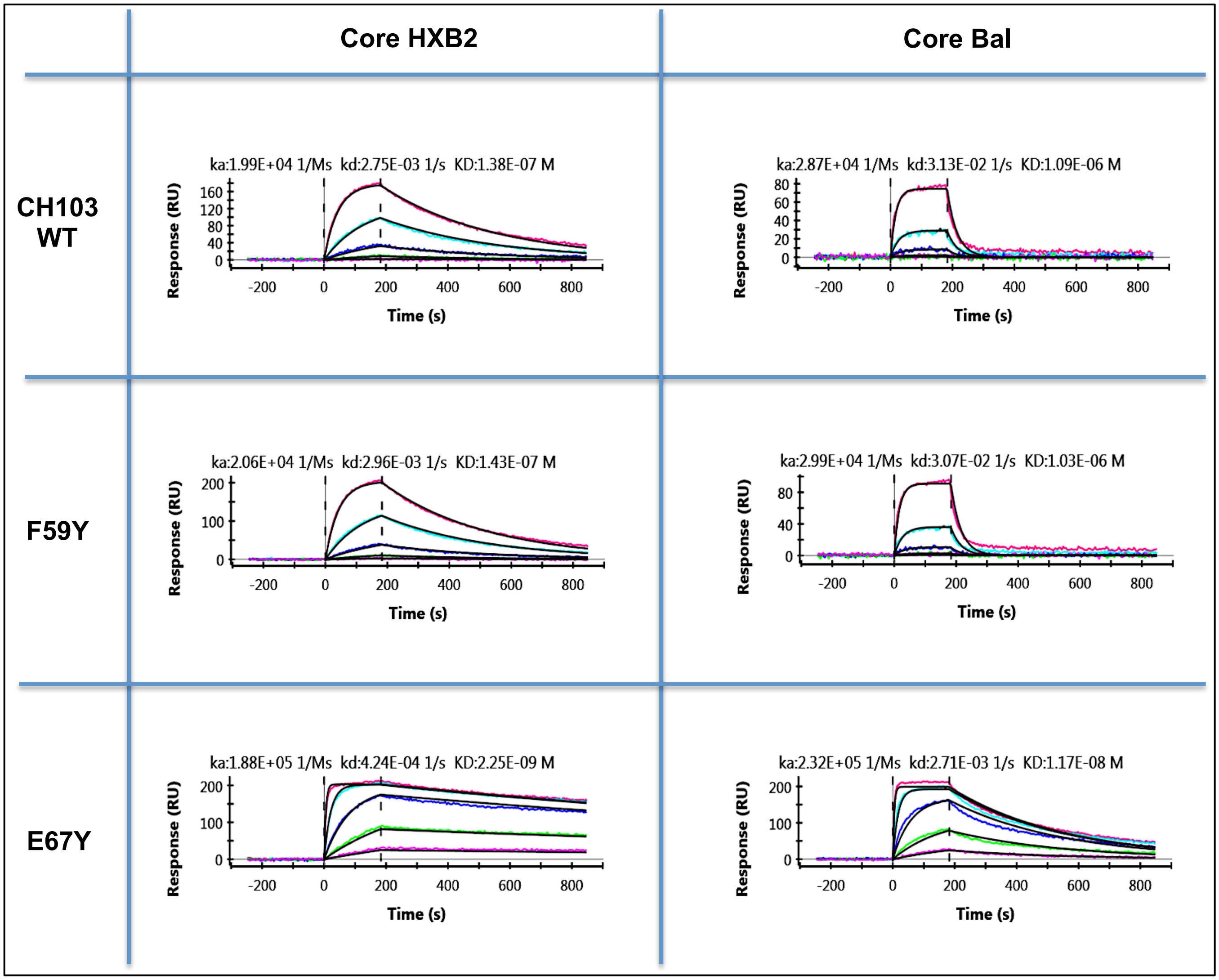

Fig. N. Kinetic Sensorgrams (ProteON XPR) of CH103 WT with mutations based on the H2_6 design.

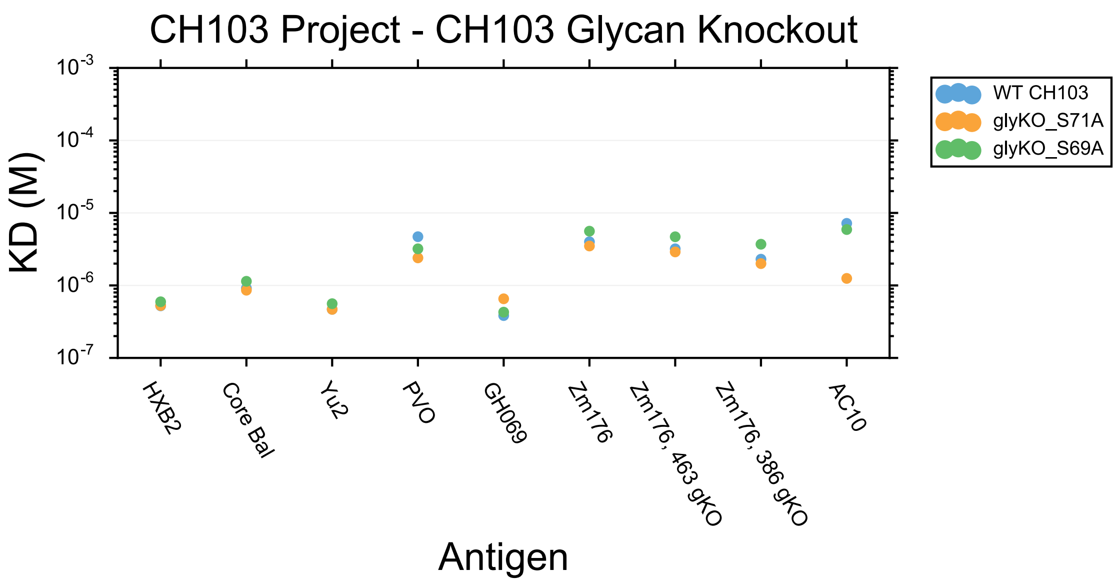

Fig. O. Paratope mutations. Two potential glycan sites were located in proximity to the antibody paratope for the CH103 antibody (PDB: 4JAN). These sites were mutated from serine to alanine mutations at position 69 and 71 in the heavy chain (AHo-Numbering). Binding affinity (K_d) is shown from Biacore experiments to nine expressed GP120s.

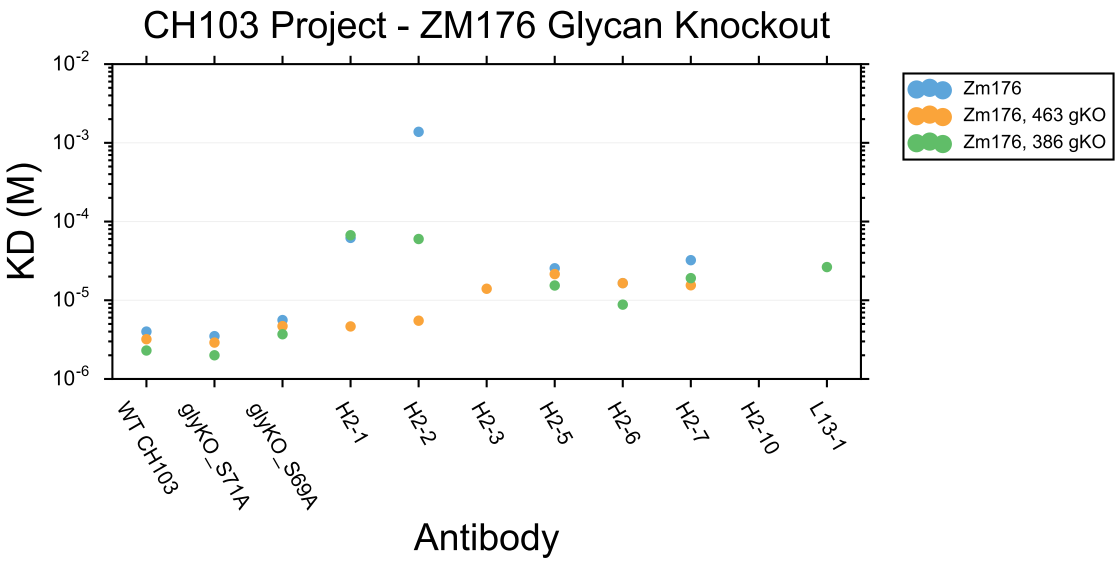

Fig. P. Epitope mutations. Potential glycan sites were located in proximity to the GP120-CH103 epitope at positions 386 and 463 of Zm176 (PDB ID 4JAN). Each of these sites were knocked out using Serine to Alanine mutations. Binding affinity (K_d) is shown from Biacore experiments to nine expressed GP120s.

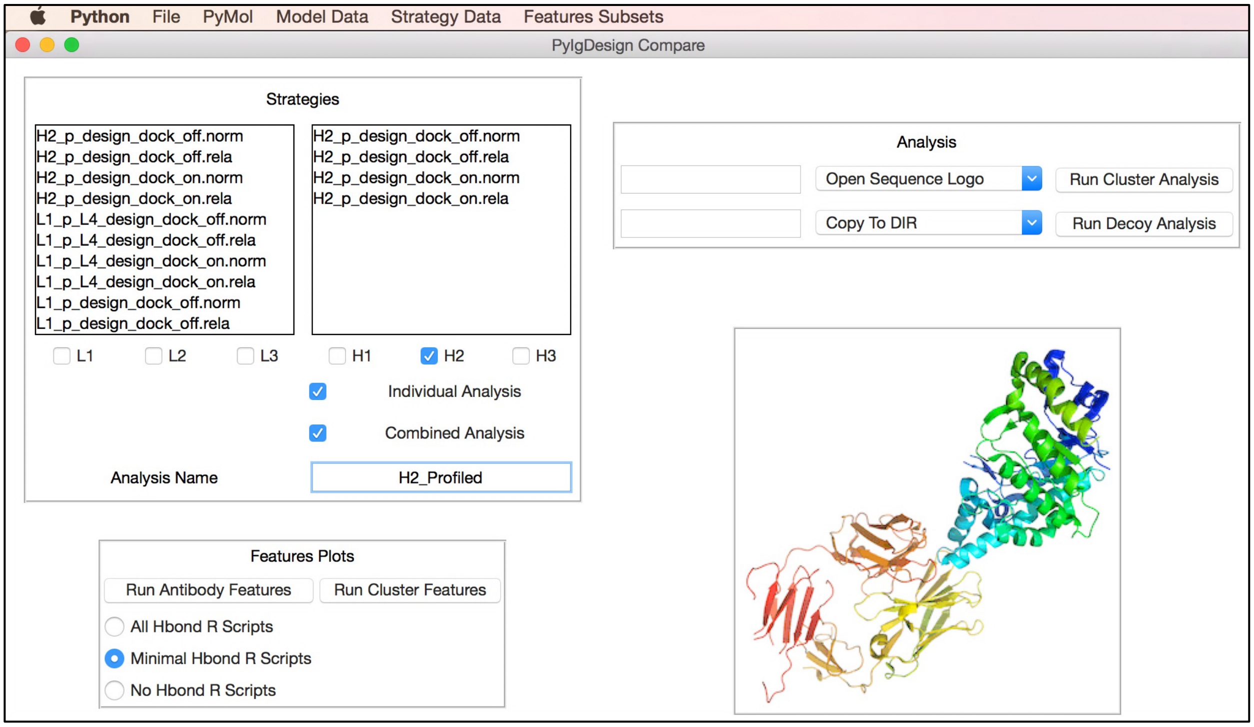

Fig. Q. Jade Antibody Design analysis Graphical User Interface (GUI)

Table A. Detailed Data on Benchmark Antibody Complexes

#	PDB	Res	Ab	VH germline	H1	H2	H3	VL Germline	L1	L2	L3	Ag	Len	Uniprot	Protein name
1	1a14	2.5	H,L	Mo_IGHV1S121	13 H1-13-1	13 H2-10-1	H3-15	Mo_IGKV10-96	14 L1-11-2	14 L2-8-1	8 L3-9-cis7-1	N	388	NRAM_I75A5(83-470)	Neuraminidase
2	1a2y	1.5	B,A	Mo_IGHV2-6-7	14 H1-13-1	5 H2-9-1	H3-10	Mo_IGKV12-41	4 L1-11-2	7 L2-8-1	2 L3-9-cis7-2	C	129	LYSC_CHICK(19-147)	Lysozyme C
3	1fe8	2.0	H,L	Mo_IGHV2-3	21 H1-13-1	9 H2-9-1	H3-9	Mo_IGKV10-96	13 L1-11-1	4 L2-8-1	12 L3-9-cis7-1	A	196	VWF_HUMAN(1683-1874)	von Willebrand factor
4	1ic7	2.1	H,L	Mo_IGHV3-8	20 H1-13-1	9 H2-9-1	H3-7	Mo_IGKV5-43	5 L1-11-1	16 L2-8-1	10 L3-9-cis7-1	Y	129	LYSC_CHICK(19-147)	Lysozyme C
5	1iqd	2.0	B,A	Hu_IGHV1-24	15 H1-13-1	5 H2-10-1	H3-10	Hu_IGKV3-20	9 L1-12-1	7 L2-8-1	55 L3-9-1	C	156	FA8_HUMAN(2193-2348)	Coagulation factor VIII
6	1n8z	2.5	B,A	Hu_IGHV3-66<br>Mo_IGHV14-3	17 H1-13-1	12 H2-10-1	H3-13	Hu_IGKV1D-39<br>Mo_IGKV6-17	10 L1-11-1	10 L2-8-1	8 L3-9-cis7-1	C	607	ERBB2_HUMAN(23-629)	ErbB-2
7	1ncb	2.5	H,L	Mo_IGHV9-3	19 H1-13-1	19 H2-10-1	H3-13	Mo_IGKV6-25	16 L1-11-2	10 L2-8-1	20 L3-9-cis7-1	N	389	NRAM_I75A5(82-470)	Neuraminidase
8	1osp	2.0	H,L	Mo_IGHV3-8	13 H1-13-7	0 H2-9-3	H3-14	Mo_IGKV13-84	9 L1-11-2	18 L2-8-2	10 L3-9-cis7-1	O	257	OSPA_BORBU(17-273)	Ozd[A
9	1uj3	2.1	B,A	Hu_IGHV1-69<br>Mo_IGHV14-1	16 H1-13-1	12 H2-10-1	H3-10	Hu_IGKV1D-16<br>Mo_IGKV14-126	5 L1-11-2	7 L2-8-1	12 L3-9-cis7-1	C	205	TF_HUMAN(38-242)	Tissue factor
10	1w72	2.2	H,L	Hu_IGHV3-9	9 H1-13-1	11 H2-10-2	H3-15	Hu_IGLV3-21	5 L1-11-3	12 L2-8-1	10 L3-11-1	A<br>B<br>C	274<br>100<br>9	1A01_HUMAN(25-298)<br>B2MG_HUMAN(21-119)<br>MAGA1_HUMAN(161-169)	HLA-A1,β2-MG,peptide
11	2adf	1.9	H,L	Mo_IGHV9-3	10 H1-13-1	13 H2-10-1	H3-11	Mo_IGKV19-93	7 L1-11-2	9 L2-8-1	7 L3-8-1	A	196	VWF_HUMAN(1683-1874)	von Willebrand factor
12	2b2x	2.2	H,L	Hu_IGHV3-66<br>Mo_IGHV5-6-5	12 H1-13-1	8 H2-9-1	H3-12	Hu_IGKV1D-13<br>Mo_IGKV4-68	13 L1-10-1	5 L2-8-1	9 L3-9-cis7-1	A	223	ITA1_RAT(151-364)	Integrin alpha-1
13	2cmr	2.0	H,L	Hu_IGHV1-69	30 H1-13-3	11 H2-10-1	H3-12	Hu_IGKV1-5	6 L1-11-1	5 L2-8-1	8 L3-9-cis7-1	A	226	D0VWW0_9HIV1(1-226)	gp41
14	2dd8	2.3	H,L	Hu_IGHV1-69	18 H1-13-10	18 H2-10-1	H3-11	Hu_IGLV3-21	7 L1-11-3	14 L2-8-1	44 L3-10-1	S	202	SPIKE_CVHSA(317-518)	Spike glycoprotein
15	2ghw	2.3	B,B	Hu_IGHV3-30	10 H1-13-1	11 H2-10-2	H3-10	Hu_IGKV3-11	12 L1-11-1	8 L2-8-1	17 L3-9-cis7-1	A	203	SPIKE_CVHSA(317-510)	Spike glycoprotein
16	2vxt	1.5	H,L	Mo_IGHV1S135	7 H1-13-1	7 H2-10-1	H3-6	Mo_IGKV9-120	9 L1-11-1	6 L2-8-1	6 L3-9-cis7-1	I	157	IL18_HUMAN(37-193)	Interleukin-18
17	2xqy	2.1	G,L	Mo_IGHV1S72	7 H1-13-1	10 H2-10-1	H3-11	Mo_IGKV3-12	10 L1-15-1	8 L2-8-1	11 L3-9-cis7-1	A	572	GH_SUHVK(107-639)	Envelope glycoprotein-H
18	2xwt	1.9	A,B	Hu_IGHV5-51	22 H1-13-1	14 H2-10-1	H3-12	Hu_IGLV1-51	9 L1-13-1	12 L2-8-2	21 L3-11-1	C	239	TSHR_HUMAN(22-260)	Thyrotropin receptor
19	2ypv	1.8	H,L	Mo_IGHV1-39	7 H1-13-1	8 H2-10-1	H3-12	Mo_IGKV14-111	7 L1-11-2	6 L2-8-1	10 L3-9-cis7-1	A	253	Q9JXV4_NEIMB(69-320)	Lipoprotein
20	3bn9	2.2	C,D	Hu_IGHV3-23	11 H1-13-1	9 H2-10-2	H3-21	Hu_IGKV1D-39	9 L1-11-1	8 L2-8-1	19 L3-9-cis7-1	B	241	ST14_HUMAN(615-855)	MT-SP1
21	3cx5	1.9	J,K	Mo_IGHV3-6	9 H1-14-1	14 H2-9-1	H3-15	Mo_IGKV10-96	11 L1-11-2	10 L2-8-1	9 L3-9-cis7-1	E	185	UCRI_YEAST(31-215)	Rieske Iron-sulfur protein
22	3ffd	2.0	A,B	Mo_IGHV5-6	9 H1-13-1	4 H2-10-2	H3-11	Mo_IGLV3	4 L1-12-3	9 L2-12-2	37 L3-13-1	P	108	PTHR_HUMAN(37-144)	PTH-related
23	3h3b	2.5	C,C	Mo_IGHV1S34	12 H1-13-1	18 H2-10-1	H3-13	Mo_IGKV8-30	7 L1-17-1	10 L2-8-1	6 L3-9-cis7-1	A	194	ERBB2_HUMAN(23-214)	ErbB-2
24	3hi6	2.3	X,Y	Hu_IGHV3-23	11 H1-13-1	22 H2-10-2	H3-13	Hu_IGKV1D-39	5 L1-11-1	11 L2-8-1	44 L3-8-1	B	180	ITAL_HUMAN(153-332)	Integrin alpha-L
25	3k2u	2.4	H,L	Hu_IGHV3-66<br>Mo_IGHV14-3	17 H1-13-1	15 H2-10-1	H3-11	Hu_IGKV1D-39<br>Mo_IGKV6-17	8 L1-11-1	9 L2-8-1	12 L3-9-cis7-1	A	257	HGFA_HUMAN(408-655)	HGF activator
26	3l95	2.2	B,A	Hu_IGHV3-74	7 H1-13-1	14 H2-10-1	H3-12	Hu_IGKV1D-39<br>Mo_IGKV6-17	8 L1-11-1	7 L2-8-1	16 L3-9-2	X	244	NOTC1_HUMAN(1448-1728)	NOTCH1
27	3mxw	1.8	H,L	Mo_IGHV1S137	9 H1-13-1	10 H2-10-1	H3-12	Mo_IGKV6-32	5 L1-11-1	7 L2-8-1	12 L3-9-cis7-1	A	169	SHH_HUMAN(29-197)	Sonic hedgehog protein
28	3nid	2.3	H,L	Mo_IGHV14-3	17 H1-13-1	7 H2-10-1	H3-12	Mo_IGKV14-100	6 L1-11-2	9 L2-8-1	5 L3-9-cis7-1	A	457	ITA2B_HUMAN(32-488)	Integrin alpha-IIb
29	3o2d	2.2	H,L	Mo_IGHV1-14	8 H1-13-1	11 H2-10-1	H3-15	Hu_IGKV4-1<br>Mo_IGKV8-30	7 L1-17-1	8 L2-8-1	7 L3-8-1	A	188	CD4_HUMAN(26-207)	CD4
30	3rkd	1.9	H,L	Mo_IGHV8-8	9 H1-15-1	13 H2-9-1	H3-16	Mo_IGKV12-46	7 L1-11-2	5 L2-8-1	13 L3-9-cis7-2	A	146	CAPSD_HEVPA(459-603)	Capsid protein
31	3s35	2.2	H,L	Mo_IGHV1-71	9 H1-13-1	13 H2-10-1	H3-10	Mo_IGKV3-5	9 L1-15-1	10 L2-8-1	5 L3-9-cis7-1	X	122	VGFR2_HUMAN(220-338)	VGFR2
32	3uzq	1.6	A,A	Mo_IGHV14-3	16 H1-13-1	9 H2-10-1	H3-9	Mo_IGKV3-5	8 L1-15-1	7 L2-8-1	26 L3-9-cis7-1	B	114	POLG_DEN1B(576-680)	Genome polyprotein
33	3w9e	2.3	A,B	Hu_IGHV1-69	29 H1-13-1	19 H2-10-1	H3-15	Hu_IGKV3-20	12 L1-12-1	11 L2-8-1	38 L3-8-2	C	306	GD_HHV2H(1-300)	Envelope glycoprotein D
34	4cmh	1.5	B,C	Mo_IGHV1-87	7 H1-13-1	9 H2-10-1	H3-13	Mo_IGKV6-17	8 L1-11-1	16 L2-8-1	23 L3-9-cis7-1	A	256	CD38_HUMAN(45-300)	CD38
35	4dtg	1.8	H,L	Hu_IGHV3-7<br>Mo_IGHV5-4	8 H1-13-1	12 H2-10-2	H3-14	Hu_IGKV2D-29<br>Mo_IGKV1-135	10 L1-16-1	9 L2-8-1	7 L3-9-cis7-1	K	66	TFPI1_HUMAN(119-178)	TFPI
36	4dvr	2.5	H,L	Hu_IGHV1-69-2	9 H1-13-1	12 H2-10-1	H3-12	Hu_IGKV1D-12	12 L1-11-1	26 L2-8-1	51 L3-8-1	G	313	ENV_HV1Y2(304-474)	gp160
37	4etq	2.1	H,L	Mo_IGHV1S37	7 H1-13-1	11 H2-10-1	H3-12	Mo_IGKV4-55	9 L1-10-1	6 L2-8-1	9 L3-9-cis7-1	C	269	Q1M1K6_9POXV(1-261)	IMV membrane protein
38	4ffv	2.4	D,C	Mo_IGHV1-39	12 H1-13-1	9 H2-10-1	H3-10	Mo_IGKV4-72	19 L1-10-1	16 L2-8-4	15 L3-9-cis7-1	B	730	DPP4_RAT(38-767)	Dipeptidyl peptidase 4
39	4fqj	2.5	H,L	Hu_IGHV1-18	22 H1-13-1	14 H2-10-1	H3-18	Hu_IGLV1-47	10 L1-13-1	14 L2-8-1	14 L3-11-1	A	304	I0B7N4_9INFB(46-343)	Hemagglutinin
40	4g6j	2.0	H,L	Hu_IGHV3-33	9 H1-13-1	10 H2-10-2	H3-11	Hu_IGKV6D-21	10 L1-11-1	10 L2-8-1	8 L3-9-cis7-1	A	158	IL1B_HUMAN(117-269)	Interleukin-1 beta

#	PDB	Res	Ab	VH germline	H1	H2	H3	VL Germline	L1	L2	L3	Ag	Len	Uniprot	Protein name
41	4g6m	1.8	H,L	Hu_IGHV4-61<br>Mo_IGHV8-8	7 H1-15-1	15 H2-9-1	H3-12	Hu_IGKV1-NL1<br>Mo_IGKV10-94	6 L1-11-2	6 L2-8-1	7 L3-9-cis7-1	A	150	IL1B_HUMAN(118-267)	Interleukin-1 beta
42	4h8w	1.8	H,L	Hu_IGHV3-23	10 H1-13-1	8 H2-10-2	H3-12	Hu_IGLV2-14	10 L1-14-2	10 L2-8-1	61 L3-11-1	G	353	Q0ED31_9HIV1(43-486)	gp160
43	4ki5	2.4	E,F	Mo_IGHV1-55	12 H1-13-1	8 H2-10-1	H3-15	Mo_IGKV9-124	9 L1-11-2	15 L2-8-2	10 L3-9-cis7-1	M	183	FA8_HUMAN(2190-2351)	Factor VIII
44	4lvn	2.3	C,B	Mo_IGHV3-1	13 H1-14-1	8 H2-9-1	H3-13	Mo_IGKV4-74	8 L1-12-1	9 L2-8-1	7 L3-9-cis7-1	A	344	Q868D6_PLAFA(330-673)	Subtilisin-like SP
45	4ot1	2.1	H,L	Hu_IGHV1-2	9 H1-13-1	11 H2-10-1	H3-24	Hu_IGLV1-51	14 L1-13-1	8 L2-8-2	54 L3-10-1	A	129	GB_HCMVT(344-438)	Envelope glycoprotein B
46	4qci	2.3	B,A	Hu_IGHV3-30	9 H1-13-1	11 H2-10-2	H3-13	Hu_IGLV3-1	10 L1-11-3	7 L2-8-1	68 L3-9-2	C<br>D	110	PDGFB_HUMAN(82-190)	PDGFR Beta
47	4xnq	2.0	B,A	Hu_IGHV4-4	39 H1-14-1	12 H2-9-1	H3-16	Hu_IGLV3-1	14 L1-11-3	9 L2-8-1	39 L3-9-1	D	212	Q6DQ33_9INFA(64-275)	Hemagglutinin (Fragment)
48	4ydk	2.1	H,L	Hu_IGHV3-23	10 H1-13-1	11 H2-10-2	H3-22	Hu_IGKV1D-33	4 L1-11-1	8 L2-8-1	23 L3-9-2	G	353	Q0ED31_9HIV1(323-484)	gp160
49	5b8c	2.1	B,A	Hu_IGHV1-18<br>Mo_IGHV1S120	9 H1-13-1	9 H2-10-1	H3-13	Hu_IGKV3-11<br>Mo_IGKV3-12	10 L1-15-1	13 L2-8-1	10 L3-9-cis7-1	C	139	PDCD1_HUMAN(32-160)	PD1
50	5bv7	2.5	C,B	Hu_IGHV3-33	16 H1-13-1	29 H2-10-2	H3-19	Hu_IGLV3-1	16 L1-11-3	16 L2-8-1	57 L3-10-1	A	422	LCAT_HUMAN(25-440)	PC-sterol acyltransferase
51	5d93	2.2	C,B	Mo_IGHV1-66	9 H1-13-1	10 H2-10-1	H3-9	Mo_IGKV4-61	5 L1-10-1	6 L2-8-1	12 L3-9-cis7-1	A	244	QSOX1_MOUSE(36-275)	Sulfhydryl oxidase 1
52	5d96	2.3	J,I	Mo_IGHV2-6-7	17 H1-13-1	7 H2-9-1	H3-12	Mo_IGKV6-17	10 L1-11-1	8 L2-8-1	11 L3-9-cis7-1	A	244	QSOX1_MOUSE(36-275)	Sulfhydryl oxidase 1
53	5en2	1.8	A,B	Mo_IGHV1-87	12 H1-13-1	10 H2-10-1	H3-17	Mo_IGKV6-13	6 L1-11-1	6 L2-8-1	8 L3-9-cis7-1	C	141	GLYC_JUNIN(87-227)	Pre-glycoprotein GP
54	5f9o	1.9	H,L	Hu_IGHV1-46	8 H1-13-1	7 H2-10-1	H3-15	Hu_IGKV3D-15	4 L1-11-1	5 L2-8-1	9 L3-8-1	G	352	A0A0M3KKW9_9HIV1(1-352)	gp120 core
55	5ggs	2.0	A,B	Hu_IGHV1-18<br>Mo_IGHV1S120	33 H1-13-3	12 H2-10-1	H3-13	Hu_IGKV3-11<br>Mo_IGKV3-12	9 L1-15-1	9 L2-8-1	13 L3-9-cis7-1	Z	123	PDCD1_HUMAN(26-148)	PD1
56	5hi4	1.8	H,L	Hu_IGHV3-23	9 H1-13-1	8 H2-10-2	H3-11	Hu_IGLV6-57	9 L1-13-2	9 L2-8-1	51 L3-9-1	A,B<br>I	132	IL17_HUMAN(24-155)	Interleukin-17A homodimer<br>+ peptide inhibitor
57	5j13	2.3	C,B	Hu_IGHV3-33	8 H1-13-1	10 H2-10-2	H3-15	Hu_IGLV3-21	10 L1-11-3	9 L2-8-2	14 L3-11-1	A	147	TSLP_HUMAN(29-159)	Thymic stromal lymphopoietin
58	5l6y	2.0	H,L	Hu_IGHV1-18	17 H1-13-1	11 H2-10-1	H3-15	Hu_IGLV3-21	7 L1-11-3	10 L2-8-1	48 L3-11-1	C	112	IL13_HUMAN(35-146)	Interleukin-13
59	5mes	2.2	H,L	Hu_IGHV3-21	11 H1-13-1	11 H2-10-2	H3-12	Hu_IGLV1-44	10 L1-13-1	8 L2-8-1	9 L3-11-1	A	162	MCL1_MOUSE(153-308)	Mcl-1 homolog
60	5nuz	1.9	A,B	Mo_IGHV1-87	7 H1-13-1	11 H2-10-1	H3-13	Mo_IGKV3-2	9 L1-15-1	8 L2-8-1	6 L3-9-cis7-1	C	156	C1K9J9_JUNIN(87-232)	Pre-glycoprotein GP

When two germlines are listed, the first is the framework and the second represents the CDRs.
For each CDR, the Pyigclassify cluster is given preceded by the distance from the cluster centroid in degrees. For H3, only the length is given.
Antibodies to the same antigen bind in different locations and are not the same antibody.

Table B. Binding affinity , CDR design identity, and selection strategies of the expressed and tested 2J88 designs from the Biacore 4000 results.

Name	Binding Affinity (M)	Dock?	Relaxed?	Selection Method	CDR	length	cluster	CDR_sequence
**Native 2J88 WT**	1.56E-08				L1	11	L1-11-1	RASENIYSYLT
**Native 2J88 WT**	1.56E-08				H2	9	H2-9-1	HIYWDDDKR
2J88_L14_7	***2.61E-09***	***dock_on***	***unrelaxed***	***dG_top_p_total***	***L1***	***11***	***L1-11-2***	***RSSRDIKDYIT***
2J88_L1_10	***3.12E-09***	***dock_on***	***unrelaxed***	***dG_top_p_total***	***L1***	***11***	***L1-11-1***	***RASKDISDYLT***
2J88_L1_5	***6.22E-09***	***dock_off***	***unrelaxed***	***delta_unsats_per_1000_dSASA***	***L1***	***11***	***L1-11-1***	***RASQDISNYLT***
2J88_H2_8	*Low Rmax*	*dock_on*	*unrelaxed*	*dG_top_p_total*	*H2*	*10*	*H2-10-1*	*AIYPSDGETR*
2J88_L14_3	*Low Rmax*	*dock_off*	*relaxed*	*dG_top_p_total*	*L1*	*15*	*L1-15-1*	*RASESVDSYGTNHIH*
2J88_L14_8	3.38E-08	dock_on	unrelaxed	dG_top_p_total	L1	11	L1-11-2	RSSRNIKDFIS
2J88_H2_4	Low Rmax	dock_off	unrelaxed	delta_unsats_per_1000_dSASA	H2	10	H2-10-2	YISWSGTVTS
2J88_L14_6	5.00E-08	dock_off	relaxed	delta_unsats_per_1000_dSASA	L1	11	L1-11-2	KSSEEIKNFIT
2J88_H2_9	5.32E-08	dock_on	relaxed	dG_top_p_total	H2	10	H2-10-2	EISSDGSRTY
2J88_L1_7	5.97E-08	dock_off	relaxed	delta_unsats_per_1000_dSASA	L1	11	L1-11-2	RASQDIKNNIT
2J88_L1_2	6.81E-08	dock_off	unrelaxed	dG_top_p_total	L1	15	L1-15-1	RASKSVDSYGSSFMS
2J88_H2_1	Low Rmax	dock_off	unrelaxed	dG_top_p_total	H2	10	H2-10-1	EIYPSDGDTR
2J88_H2_5	Low Rmax	dock_off	unrelaxed	delta_unsats_per_1000_dSASA	H2	10	H2-10-1	AIYGEDGETR
2J88_L1_11	2.02E-07	dock_on	relaxed	dG_top_p_total	L1	15	L1-15-1	RASKSVDSYGFSFMS
2J88_L14_9	2.76E-07	dock_on	relaxed	dG_top_p_total	L1	11	L1-11-2	RSSEDIKNFIS
2J88_L1_9	4.30E-07	dock_on	unrelaxed	dG_top_p_total	L1	11	L1-11-1	RASKDISKYIA
2J88_L1_1	4.45E-07	dock_off	unrelaxed	dG_top_p_total	L1	15	L1-15-1	RASESVESYGSSFIS
2J88_L1_6	7.69E-07	dock_off	unrelaxed	delta_unsats_per_1000_dSASA	L1	11	L1-11-1	QASQDVGDALT
2J88_L14_1	Low Rmax	dock_off	unrelaxed	dG_top_p_total	L1	17	L1-17-1	KSSHSLLNSSAQMNYLS
2J88_L1_4	1.38E-06	dock_off	relaxed	dG_top_p_total	L1	17	L1-17-1	KSSQSVLNSRSEKSYLT
2J88_L1_12	1.58E-06	dock_on	relaxed	dG_top_p_total	L1	15	L1-15-1	RASKSVESYGNSFIS
2J88_L14_4	1.59E-06	dock_off	unrelaxed	delta_unsats_per_1000_dSASA	L1	11	L1-11-1	RASQIVSYALS
2J88_L1_8	2.19E-06	dock_off	relaxed	delta_unsats_per_1000_dSASA	L1	15	L1-15-1	RASESVDSYGNSFIS
2J88_L1_3	2.38E-06	dock_off	relaxed	dG_top_p_total	L1	15	L1-15-1	RASESVESYGNSFMH
2J88_L14_2	6.21E-06	dock_off	unrelaxed	dG_top_p_total	L1	17	L1-17-1	KSSQSLLNSNAEKNYLT
2J88_H2_3	9.47E-06	dock_off	relaxed	dG_top_p_total	H2	12	H2-12-1	EIRSKADGSATH
2J88_L14_5	0	dock_off	unrelaxed	delta_unsats_per_1000_dSASA	L1	11	L1-11-2	RSSRDIKNAIS
2J88_H2_6	0	dock_off	relaxed	delta_unsats_per_1000_dSASA	H2	10	H2-10-6	WINLDGGSTS
2J88_H2_2	0	dock_off	unrelaxed	dG_top_p_total	H2	12	H2-12-1	EIGSKSFGGETK
2J88_H2_7	0	dock_on	unrelaxed	dG_top_p_total	H2	10	H2-10-1	MIYPSDGDTR

Table C. Binding affinity of expressed and purified CH103 designs and their selection strategies.

**Name**	**Antigen**	**Binding Affinity (M)**	by_score_group	strategy
**Native**	HXB2	5.23E-07		
glyKO_S71A	HXB2	5.28E-07		
glyKO_S69A	HXB2	5.95E-07		
H2-1	HXB2	6.70E-06	dG_top_Ptotal	ch103_H2_all_p.norm
H2-2	HXB2	3.70E-06	dG_top_Ptotal	ch103_H2_all_p.norm
H2-3	HXB2	3.70E-06	dG_top_Ptotal	ch103_H2_all_p.norm
H2-4	HXB2	0	dG_top_Ptotal	ch103_H2_all.rela
**H2-5**	HXB2	**5.16E-07**	dG_top_Ptotal	ch103_H2_all.rela
H2-6	HXB2	***1.32E-08***	delta_unsats_per_1000_dSASA	ch103_H2_all_p.rela
**H2-7**	HXB2	**8.92E-07**	delta_unsats_per_1000_dSASA	ch103_H2_all_p.rela
H2-8	HXB2	0	delta_unsats_per_1000_dSASA	ch103_H2_all_p.rela
H2-9	HXB2	0	sc_value	ch103_H2_all_p.rela
H2-10	HXB2	1.67E-05	sc_value	ch103_H2_all.rela
L13-1	HXB2	0	dG_top_Ptotal	ch103_L1L3_all_p.norm
**Native**	Core Bal	**9.15E-07**		
glyKO_S71A	Core Bal	8.59E-07		
glyKO_S69A	Core Bal	1.14E-06		
H2-1	Core Bal	1.14E-05	dG_top_Ptotal	ch103_H2_all_p.norm
H2-2	Core Bal	7.00E-06	dG_top_Ptotal	ch103_H2_all_p.norm
H2-3	Core Bal	6.00E-06	dG_top_Ptotal	ch103_H2_all_p.norm
H2-4	Core Bal	0.00E+00	dG_top_Ptotal	ch103_H2_all.rela
**H2-5**	Core Bal	1.20E-06	dG_top_Ptotal	ch103_H2_all.rela
H2-6	Core Bal	***1.69E-08***	delta_unsats_per_1000_dSASA	ch103_H2_all_p.rela
**H2-7**	Core Bal	2.40E-06	delta_unsats_per_1000_dSASA	ch103_H2_all_p.rela
H2-8	Core Bal	0	delta_unsats_per_1000_dSASA	ch103_H2_all_p.rela
H2-9	Core Bal	0	sc_value	ch103_H2_all_p.rela
H2-10	Core Bal	5.50E-06	sc_value	ch103_H2_all.rela
L13-1	Core Bal	0	dG_top_Ptotal	ch103_L1L3_all_p.norm
**Native**	Yu2	**4.68E-07**		
glyKO_S71A	Yu2	4.68E-07		
glyKO_S69A	Yu2	5.60E-07		
H2-1	Yu2	0	dG_top_Ptotal	ch103_H2_all_p.norm
H2-2	Yu2	1.18E-05	dG_top_Ptotal	ch103_H2_all_p.norm
H2-3	Yu2	5.30E-06	dG_top_Ptotal	ch103_H2_all_p.norm
H2-4	Yu2	0	dG_top_Ptotal	ch103_H2_all.rela
**H2-5**	Yu2	6.10E-06	dG_top_Ptotal	ch103_H2_all.rela
H2-6	Yu2	***7.24E-08***	delta_unsats_per_1000_dSASA	ch103_H2_all_p.rela
**H2-7**	Yu2	3.70E-06	delta_unsats_per_1000_dSASA	ch103_H2_all_p.rela
H2-8	Yu2	0	delta_unsats_per_1000_dSASA	ch103_H2_all_p.rela
H2-9	Yu2	0	sc_value	ch103_H2_all_p.rela
H2-10	Yu2	6.50E-06	sc_value	ch103_H2_all.rela
L13-1	Yu2	0	dG_top_Ptotal	ch103_L1L3_all_p.norm
**Native**	PVO	**4.70E-06**		
glyKO_S71A	PVO	2.40E-06		
glyKO_S69A	PVO	3.20E-06		
H2-1	PVO	7.70E-05	dG_top_Ptotal	ch103_H2_all_p.norm
H2-2	PVO	0	dG_top_Ptotal	ch103_H2_all_p.norm
H2-3	PVO	9.20E-06	dG_top_Ptotal	ch103_H2_all_p.norm
H2-4	PVO	0	dG_top_Ptotal	ch103_H2_all.rela
**H2-5**	PVO	5.80E-06	dG_top_Ptotal	ch103_H2_all.rela
H2-6	PVO	**3.50E-07**	delta_unsats_per_1000_dSASA	ch103_H2_all_p.rela
**H2-7**	PVO	5.40E-06	delta_unsats_per_1000_dSASA	ch103_H2_all_p.rela
H2-8	PVO	0	delta_unsats_per_1000_dSASA	ch103_H2_all_p.rela
H2-9	PVO	0	sc_value	ch103_H2_all_p.rela
H2-10	PVO	0	sc_value	ch103_H2_all.rela
L13-1	PVO	0	dG_top_Ptotal	ch103_L1L3_all_p.norm

**Name**	**Antigen**	**Binding Affinity (M)**	by_score_group	strategy
**Native**	GH069	3.84E-07		
glyKO_S71A	GH069	6.54E-07		
glyKO_S69A	GH069	4.28E-07		
H2-1	GH069	0	dG_top_Ptotal	ch103_H2_all_p.norm
H2-2	GH069	1.18E-06	dG_top_Ptotal	ch103_H2_all_p.norm
H2-3	GH069	5.97E-07	dG_top_Ptotal	ch103_H2_all_p.norm
H2-4	GH069	0	dG_top_Ptotal	ch103_H2_all.rela
**H2-5**	GH069	5.98E-07	dG_top_Ptotal	ch103_H2_all.rela
H2-6	GH069	**1.42E-07**	delta_unsats_per_1000_dSASA	ch103_H2_all_p.rela
**H2-7**	GH069	2.70E-06	delta_unsats_per_1000_dSASA	ch103_H2_all_p.rela
H2-8	GH069	0	delta_unsats_per_1000_dSASA	ch103_H2_all_p.rela
H2-9	GH069	0	sc_value	ch103_H2_all_p.rela
H2-10	GH069	0	sc_value	ch103_H2_all.rela
L13-1	GH069	0	dG_top_Ptotal	ch103_L1L3_all_p.norm
**Native**	Zm176	**4.00E-06**		
glyKO_S71A	Zm176	3.50E-06		
glyKO_S69A	Zm176	5.60E-06		
H2-1	Zm176	6.20E-05	dG_top_Ptotal	ch103_H2_all_p.norm
H2-2	Zm176	1.38E-03	dG_top_Ptotal	ch103_H2_all_p.norm
H2-3	Zm176	0	dG_top_Ptotal	ch103_H2_all_p.norm
H2-4	Zm176	0	dG_top_Ptotal	ch103_H2_all.rela
**H2-5**	Zm176	2.55E-05	dG_top_Ptotal	ch103_H2_all.rela
H2-6	Zm176	1.65E-05	delta_unsats_per_1000_dSASA	ch103_H2_all_p.rela
**H2-7**	Zm176	3.23E-05	delta_unsats_per_1000_dSASA	ch103_H2_all_p.rela
H2-8	Zm176	0	delta_unsats_per_1000_dSASA	ch103_H2_all_p.rela
H2-9	Zm176	0	sc_value	ch103_H2_all_p.rela
H2-10	Zm176	0	sc_value	ch103_H2_all.rela
L13-1	Zm176	0	dG_top_Ptotal	ch103_L1L3_all_p.norm
**Native**	Zm176, 463 gKO	**3.20E-06**		
glyKO_S71A	Zm176, 463 gKO	2.90E-06		
glyKO_S69A	Zm176, 463 gKO	4.69E-06		
H2-1	Zm176, 463 gKO	**4.65E-06**	dG_top_Ptotal	ch103_H2_all_p.norm
H2-2	Zm176, 463 gKO	**5.50E-06**	dG_top_Ptotal	ch103_H2_all_p.norm
H2-3	Zm176, 463 gKO	1.40E-05	dG_top_Ptotal	ch103_H2_all_p.norm
H2-4	Zm176, 463 gKO	0	dG_top_Ptotal	ch103_H2_all.rela
**H2-5**	Zm176, 463 gKO	2.15E-05	dG_top_Ptotal	ch103_H2_all.rela
H2-6	Zm176, 463 gKO	1.65E-05	delta_unsats_per_1000_dSASA	ch103_H2_all_p.rela
**H2-7**	Zm176, 463 gKO	1.55E-05	delta_unsats_per_1000_dSASA	ch103_H2_all_p.rela
H2-8	Zm176, 463 gKO	0	delta_unsats_per_1000_dSASA	ch103_H2_all_p.rela
H2-9	Zm176, 463 gKO	0	sc_value	ch103_H2_all_p.rela
H2-10	Zm176, 463 gKO	0	sc_value	ch103_H2_all.rela
L13-1	Zm176, 463 gKO	**0**	dG_top_Ptotal	ch103_L1L3_all_p.norm
**Native**	Zm176, 386 gKO	**2.30E-06**		
glyKO_S71A	Zm176, 386 gKO	2.00E-06		
glyKO_S69A	Zm176, 386 gKO	3.70E-06		
H2-1	Zm176, 386 gKO	6.70E-05	dG_top_Ptotal	ch103_H2_all_p.norm
H2-2	Zm176, 386 gKO	6.00E-05	dG_top_Ptotal	ch103_H2_all_p.norm
H2-3	Zm176, 386 gKO	0	dG_top_Ptotal	ch103_H2_all_p.norm
H2-4	Zm176, 386 gKO	0	dG_top_Ptotal	ch103_H2_all.rela
**H2-5**	Zm176, 386 gKO	1.54E-05	dG_top_Ptotal	ch103_H2_all.rela
H2-6	Zm176, 386 gKO	**8.80E-06**	delta_unsats_per_1000_dSASA	ch103_H2_all_p.rela
**H2-7**	Zm176, 386 gKO	1.91E-05	delta_unsats_per_1000_dSASA	ch103_H2_all_p.rela
H2-8	Zm176, 386 gKO	0	delta_unsats_per_1000_dSASA	ch103_H2_all_p.rela
H2-9	Zm176, 386 gKO	0	sc_value	ch103_H2_all_p.rela
H2-10	Zm176, 386 gKO	0	sc_value	ch103_H2_all.rela
L13-1	Zm176, 386 gKO	**2.64E-05**	dG_top_Ptotal	ch103_L1L3_all_p.norm
**Native**	AC10	**7.18E-06**		
glyKO_S71A	AC10	1.25E-06		
glyKO_S69A	AC10	5.90E-06		
H2-1	AC10	0	dG_top_Ptotal	ch103_H2_all_p.norm
H2-2	AC10	0	dG_top_Ptotal	ch103_H2_all_p.norm
H2-3	AC10	0	dG_top_Ptotal	ch103_H2_all_p.norm
H2-4	AC10	0	dG_top_Ptotal	ch103_H2_all.rela
**H2-5**	AC10	0	dG_top_Ptotal	ch103_H2_all.rela
H2-6	AC10	**4.26E-06**	delta_unsats_per_1000_dSASA	ch103_H2_all_p.rela
**H2-7**	AC10	0	delta_unsats_per_1000_dSASA	ch103_H2_all_p.rela
H2-8	AC10	0	delta_unsats_per_1000_dSASA	ch103_H2_all_p.rela
H2-9	AC10	0	sc_value	ch103_H2_all_p.rela
H2-10	AC10	0	sc_value	ch103_H2_all.rela
L13-1	AC10	0	dG_top_Ptotal	ch103_L1L3_all_p.norm

Table D. *CDRClusterFeatures* reporter tables

Column	Description
	Table cdr_clusters
struct_id	Structure number – used to query other tables for same loaded structure
cdr_start	Starting number of CDR
cdr_end	Ending number of CDR
chain	Chain of CDR – L or H
CDR	Identity of CDR
length	Length of CDR
cluster	Identified cluster of CDR
dis	Calculated Dihedral Distance to cluster center
normDis	Dihedral Distance normalized by length (radians)
normDis_deg	Dihedral Distance normalized by length (degrees)
sequence	Amino acid sequence of the CDR

Table E. *InterfaceFeature* reporter tables

Column	Description
	Table interfaces
struct_id	Structure number – used to query other tables for same loaded structure
interface	Interface ID – The interface that is being analyzed. The ID lists the chains that make up the interface - such as L_A or LH_A. Used to query other tables
chains_side1	Interface Side 1 ID – chains that make up interface side 1 such as L or LH. Used to query other tables
chains_side2	Same as above, but for side 2
nchains_side1	Number of chains that make up side 1
nchains_side2	Number of chains that make up side 2
dSASA	Difference in Buried Solvent Accessible Surface Area as described above *(SASA separated - SASA together)*
dSASA_hphobic	Hydrophobic component of dSASA
dSASA_polar	Polar component of dSASA
dG	Difference in Rosetta energy as described above *(E together - E separated)*
dG_cross	Total cross interface energy of pair-wise terms. Close to dG.
dG_dev_dSASAx100	dG normalized by dSASA. Roughly an energy density calculation.
dG_cross_dev_dSASAx100	dG_cross normalized by dSASA. Roughly an energy density calculation.
hbond_E_fraction	Fraction of dG attributable to cross interface hydrogen bonds.
sc_value	Shape Complementarity value
packstat	Packing quality measure
nres_int	Total interface residues
nres_all	Total structure residues
complex_normalized	Total energy/Total residues
	Table interface_sides
struct_id	Structure number – used to query other tables for same loaded structure
interface	Interface ID – The interface that is being analyzed. The ID lists the chains that make up the interface - such as L_A or LH_A. Used to query other tables
side	The Side that this data pertains to. side1 or side2.
chains_side1	Interface Side 1 ID – chains that make up interface side 1 such as L or LH. Used to query other tables
chains_side2	Same as above, but for side 2
interface_nres	Total number of residues for interface side
dSASA	dSASA of interface side
dSASA_sc	dSASA of the side chains of the interface side
dhSASA	Hydrophobic dSASA of the interface side

dhSASA_sc	Hydrophobic dSASA of the side chains of the interface side
dG	dG of the interface side
energy_int	Energy of the interface residues complexed
energy_sep	Energy of the interface residues separated
avg_per_residue_energy_dG	Average per-residue dG of the side
avg_per_residue_energy_int	Average per-residue energy of the side while complexed
avg_per_residue_energy_sep	Average per-residue energy of the side while separated
avg_per_residue_dSASA	Average per-residue dSASAS of the side
avg_per_residue_SASA_int	Average per-residue total SASA of the side while complexed
avg_per_residue_SASA_sep	Average per-residue total SASA of the side while separated
aromatic_fraction	Fraction of total residues that are aromatic
aromatic_dSASA_fraction	Fraction of aromatic dSASA from total dSASA of the side
aromatic_dG_fraction	Fraction of aromatic dG from total dG of the side
interface_to_surface_fraction	Fraction of side interface residues to total surface residues
ss_sheet_fraction	Fraction of side interface residues that are identified as beta-sheet from DSSP
ss_helix_fraction	Fraction of side interface residues that are identified as helix from DSSP
ss_loop_fraction	Fraction of side interface residues that are identified as loop from DSSP
Table interface_residues	
struct_id	Structure number – used to query other tables for same loaded structure
interface	Interface ID – The interface that is being analyzed. The ID lists the chains that make up the interface - such as L_A or LH_A. Used to query other tables
resNum	Residue number of particular residue for which this data reports.
chains_side1	Interface Side 1 ID – chains that make up interface side 1 such as L or LH. Used to query other tables
chains_side2	Same as above, but for side 2
side	The Side that this residue is part of. Side1 or side2.
dSASA	dSASA of the residue
dSASA_sc	dSASA of the side-chain of the residue
dhSASA	Hydrophobic dSASA of the residue
dhSASA_sc	Hydrophobic dSASA of the side-chain of the residue
SASA_int	Total SASA of the residue while complexed
SASA_sep	Total SASA of the residue while separated
relative_dSASA_fraction	Fraction of dSASA of the residue relative to the total SASA separated. 0 indicates no burial, while 1 indicates total burial.
dG	dG of the residue
energy_int	energy of the residue while complexed
energy_sep	energy of the residue while separated

Table F. Additional *AntibodyFeature* reporter tables

Column	Description
Table ab_metrics	
struct_id	Structure number – used to query other tables for same loaded structure
numbering_scheme	The numbering scheme used to define the antibody
cdr_definition	The CDR Definition used to define the start and end points of the CDR
cdr_residues	The total number of CDR residues
antigen_present	Is an antigen present?
antigen_chains	If so, what are its chains?
net_charge	Net charge of the antibody
paratope_charge	Net charge of the paratope
paratope_SASA	Total SASA of the paratope
paratope_hSASA	Total Hydrophobic SASA of the paratope
paratope_pSASA	Total Polar SASA of the paratope
VL_VH_packing_angle	VH/VL Packing Angle
VL_VH_distance	VH/VL Distance
VL_VH_opening_angle	VH/VL Opening Angle
VL_VH_opposite_opening_angle	VH/VL Opposite Opening Angle

is_camelid	Is the antibody a Camelid antibody?
Table cdr_metrics	
struct_id	Structure number – used to query other tables for same loaded structure
CDR	The identity of the CDR
length	The length of the CDR
start	Starting resNum of the CDR
end	Ending resNum of the CDR
ag_ab_contacts_total	Total unique Antigen/Antibody contacts from this CDR. An atomic contact is defined as at least 5 atoms of the Ag that are within 5 Å of an Ab atom
ag_ab_contacts_nres	Total number of residues making contact with Antigen
ag_ab_dSASA	dSASA of CDR/Antigen interface
ag_ab_dSASA_sc	dSASA of only side chains of CDR/Antigen interface
ag_ab_dhSASA	Hydrophobic dSASA of CDR/Antigen interface
ag_ab_dhSASA_sc	Hydrophobic dSASA of only side chains of CDR/Antigen interface
ag_ab_dG	dG of CDR/Antigen interface
SASA	Total SASA of the CDR
charge	Total charge of the CDR
energy	Total energy of the CDR
anchor_CN_distance	Distance from C of first residue to N of last residue of the CDR
aromatic_nres	Total aromatic residues of this CDR
Table cdr_residues	
struct_id	Structure number – used to query other tables for same loaded structure
resNum	Residue number of particular residue for which this data reports.
CDR	The CDR for which this residue belongs
position	The position (1 – length) for which this is in the CDR
ag_contacts	The total number of Antigen contacts this residue makes as described above.
Table ab_h3_kink_metrics	
struct_id	Structure number – used to query other tables for same loaded structure
kink_type	The identified Kink type – either Kinked or Extended.
begin	Start of the kink
end	End of the kink
anion_res	Identity of the anion residue of the Kink
cation_res	Identify of the cation residue of the Kink
RD_Hbond_dis	Distance of the sc-sc Hbond across the strands at the beginning of the H3 kink (typically Asp-Arg)
bb_Hbond_dis	Deturns of the bb-bb Hbond across the strands at the beginning of the kink (typically Asp-Arg)
Trp_Hbond_dis	Distance of the Trp sc-bb Hbond across the H3 kink residues (n-1 to n+2)
qdis	Distance from the four kink residues of the H3 C-terminal end that make up the qdih below.
qdih	Dihedral angle from the four kink residues of the H3 C-terminal end
